# Ergosterol: Biological Activities, Mechanistic Evidence, Pharmacokinetic Barriers, and Delivery Strategies

**DOI:** 10.3390/ijms27104198

**Published:** 2026-05-08

**Authors:** Mingkai Yao, Cui Li, Mengya Dang, Na Zhang, Xiaoyun Yang, Yuping Wang, Mengru Cai, Dong Bai

**Affiliations:** Institute of Basic Theory for Chinese Medicine, China Academy of Chinese Medical Sciences, Beijing 100700, China; yaomingkai2002@163.com (M.Y.); 16696322685@163.com (C.L.); mengyad2024@163.com (M.D.); bucmzn@163.com (N.Z.); yxyhappy1999@163.com (X.Y.); wangyupingwyp@icloud.com (Y.W.)

**Keywords:** ergosterol, bioactivity, pharmacokinetics, bioavailability, delivery systems

## Abstract

Ergosterol is a fungal sterol with broad pharmacological activities, including anticancer, anti-inflammatory, cholesterol- and uric acid-lowering effects, and serves as the precursor of vitamin D_2_. Despite its therapeutic potential, poor aqueous solubility, physicochemical instability, and low oral bioavailability severely limit its clinical application. This review provides an integrative overview of the physicochemical properties, biosynthesis, biological activities, underlying molecular mechanisms, pharmacokinetics, and safety profiles of ergosterol and its major derivatives. A structured literature search was conducted in PubMed, Web of Science, Scopus and CNKI up to February 2026, with findings critically synthesized across preclinical models. Current evidence links ergosterol-related interventions to changes in signaling pathways such as PI3K/Akt, NF-κB, and Wnt/β-catenin, although direct target-engagement evidence remains limited for many disease models. The review concludes that while ergosterol represents a promising natural scaffold for drug development, translational progress is constrained by limited human pharmacokinetic data and insufficient exposure–response validation. Future research should prioritize metabolite profiling, clinically relevant dosing strategies, and formulation optimization to better define the translational potential of ergosterol-based compounds.

## 1. Introduction

Ergosterol is a fungal sterol that has attracted increasing attention as a bioactive natural compound, a precursor of vitamin D_2_, a fungal biomass marker, and a potential scaffold for pharmacological development. Preclinical studies have associated ergosterol and ergosterol-related compounds with anticancer [1], anti-inflammatory [2], metabolic regulatory [3], cholesterol-lowering [4], uric acid-lowering [5], neuroprotective [6], and organ-protective activities [7]. These findings indicate broad biological relevance, but they also create an important interpretative challenge: different studies often involve different active entities, including parent ergosterol, ergosterol peroxide, brassicasterol, other metabolites, or ergosterol-containing extracts. This review primarily focuses on parent ergosterol, while representative ergosterol-derived compounds, particularly ergosterol peroxide (EP), are also considered when discussing disease-related biological activities.

Despite growing interest, the translational value of ergosterol remains uncertain. Parent ergosterol has poor aqueous solubility [8], physicochemical instability [9], limited oral absorption [10], extensive metabolic conversion [11], and predominant fecal elimination [12]. Mechanistic interpretation also remains challenging. Although many studies report changes in PI3K/Akt [13], NF-κB [2], Wnt/β-catenin [14], LXR [15], MAPK [16], or STAT3 signaling [17], direct ligand–target evidence for ergosterol is still limited in most disease models. Therefore, the biological effects of ergosterol-related compounds should be interpreted together with compound identity, pharmacokinetic exposure, disease model, and mechanistic certainty.

This review focuses on the translational development of ergosterol rather than providing a catalogue of biological activities. We first summarize its chemical and biological characteristics, including source distribution, fungal membrane function, vitamin D_2_ formation, and analytical use as a fungal biomass marker. We then discuss pharmacokinetic barriers, active-entity attribution, and disease-oriented preclinical evidence. Finally, we critically evaluate formulation and delivery strategies and identify key gaps that should be addressed before ergosterol-based compounds can be advanced as functional ingredients, drug leads, formulation excipients, or scaffolds for derivative development.

## 2. Literature Search Strategy

This review used a structured literature search and narrative synthesis approach to summarize the chemical characteristics, biological functions, pharmacokinetic limitations, active-entity attribution, and delivery systems of ergosterol and related compounds. Literature searches were conducted in PubMed, Web of Science, Scopus, and CNKI from database inception to February 2026. Search terms included “ergosterol”, “ergosterol peroxide”, “brassicasterol”, “fungal sterol”, “vitamin D_2_ precursor”, “pharmacokinetics”, “ADME”, “absorption”, “bioavailability”, “metabolism”, “metabolite”, “delivery system”, “liposome”, “nanoparticle”, “microemulsion”, “micelle”, “ferritin cage”, “anticancer”, “anti-inflammatory”, “diabetes”, “cholesterol”, “uric acid”, “neuroprotection”, and “gut microbiota”. Boolean combinations were applied where appropriate, such as “ergosterol AND pharmacokinetics”, “ergosterol AND bioavailability”, “ergosterol peroxide AND cancer”, “ergosterol AND delivery system”, and “ergosterol AND metabolite”.

Eligible studies included original research articles and relevant reviews focusing on ergosterol, ergosterol peroxide, brassicasterol, or other ergosterol-related metabolites. Priority was given to studies reporting chemical structure and physicochemical properties, biosynthesis and natural distribution, fungal membrane function, vitamin D_2_ formation, pharmacokinetics, absorption and metabolism, disease-related bioactivity, molecular mechanisms, delivery systems, formulation characteristics, safety, or translational limitations. *In vitro* studies, animal studies, analytical method studies, formulation studies, and mechanistic studies relevant to translational development were considered eligible. Studies were excluded if they were not directly related to ergosterol or ergosterol-related compounds, only repeated general background information without extractable data, lacked accessible full text, were non-peer-reviewed sources, or had limited relevance to the scope of this review.

Records identified through the searches were first screened by title and abstract, followed by full-text assessment for relevance. For topics with multiple related studies, preference was given to articles with clearer experimental design, more complete mechanistic information, more detailed pharmacokinetic or formulation parameters, or stronger relevance to translational development. Extracted information included compound type, experimental model, dose or formulation parameters, major biological effects, molecular mechanisms, pharmacokinetic indicators, delivery-system characteristics, and key limitations. Because the included studies differed substantially in compound form, dose, model system, endpoint selection, and experimental design, no meta-analysis was performed. Instead, findings were synthesized narratively in a structured manner, with particular emphasis on active-entity attribution among parent ergosterol, ergosterol peroxide, and metabolites; whether pharmacokinetic exposure supports the interpretation of disease-model efficacy; and whether delivery systems provide feasible strategies for translational development of ergosterol-based compounds. No language restriction was applied during the initial search; however, only studies with accessible full texts and sufficient extractable information were included in the final synthesis. Duplicate records retrieved from different databases were removed before title/abstract screening.

## 3. Chemical and Biological Characteristics of Ergosterol

Ergosterol is a steroidal compound containing a cyclopentanoperhydrophenanthrene nucleus. Similar to other sterols, it has low polarity and poor aqueous solubility. The conjugated double-bond system in the B ring increases its chemical reactivity and susceptibility to oxidation. Under ultraviolet irradiation, it can be converted to vitamin D_2_. It is also prone to thermal degradation. Ergosterol occurs mainly in fungi but is also present in some protists. Ergosterol biosynthesis follows two distinct pathways. In most fungi, lanosterol is the precursor via the Δ24(28)-alkene pathway. In some protists, cycloartenol serves as the precursor in a Δ25(27)-alkene pathway, similar to cholesterol synthesis in higher plants.

### 3.1. Chemical Structure and Physicochemical Properties

Ergosterol is a sterol compound with low polarity and low water solubility. The B ring of ergosterol contains two conjugated double bond systems at Δ^5^ and Δ^7^, which makes its chemical properties more reactive. Ergosterol can form a cholesterol-like crystal structure in aqueous environments. Its molecules are arranged in a compact bilayer-like structure, similar to a phospholipid bilayer, with hydrophilic and hydrophobic layers. This makes it a component of fungal cell membranes. The conjugated system in ergosterol increases its susceptibility to oxidation under UV exposure. It is also prone to degradation at higher temperatures. Ergosterol can be oxidized after prolonged exposure to air.

#### 3.1.1. Chemical Structural Features

**Figure 1:**
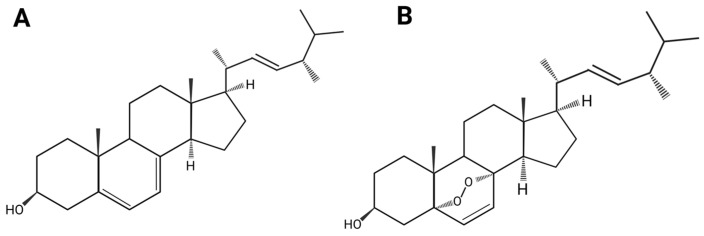
Structural diagram of ergosterol and its derivatives. (**A**) shows the chemical structure of ergosterol; (**B**) shows the structure of ergosterol peroxide.

Ergosterol is a sterol compound with the chemical formula C28H44O. Its basic structure is a cyclopentane polyhydrophenanthrene skeleton, shown in Figure 1A. A hydroxyl group is at carbon 3. A methyl group is at positions 10 and 13. Position 17 contains a 9-carbon unsaturated side chain. A distinguishing structural feature of ergosterol is its conjugated diene system involving the Δ^5^ and Δ^7^ double bonds in the B ring. There is a Δ^22^ trans double bond between C-22 and C-23 on the side chain [18]. The hydrogen atoms on C-20 are in the α configuration, and the methyl group on C-24 is in the β configuration (24β-CH_3_). The C-22 double bond is trans to C-13. The whole side chain tends to adopt a right-handed favored conformation [18]. This structural feature distinguishes ergosterol from other plant or animal sterols and helps identify species that contain it. Ergosterol can form a cholesterol-like crystalline structure in aqueous environments. Crystallographic studies of its fluorescent analogue, dehydroergosterol (DHE), show that it crystallizes in the monoclinic system (space group P2_1_). Here, molecules are in a compact bilayer-like arrangement [19]. This structure provides a physicochemical foundation for understanding membrane biological functions. Its amphiphilic nature and tendency toward tight packing determine its ability to modulate membrane order and microdomain formation in lipid bilayers. Its specific three-dimensional form also provides a basis for interactions with proteins such as steroid carrier protein-2 (SCP-2). These precise molecular and self-assembly properties form the basis for understanding its membrane functions and interactions with carrier proteins [19].

#### 3.1.2. Physicochemical Properties of Ergosterol

Ergosterol is a common sterol compound, usually a white to pale yellow crystalline powder or solid at room temperature and pressure. Ergosterol is neutral in terms of acidity and basicity, with low polarity [20,21]. It is lipophilic, soluble in organic solvents such as chloroform, methanol, and hexane [8,21,22,23,24,25], and exhibits good solubility and extraction efficiency in natural deep eutectic solvents (NADES) [8]. Therefore, ergosterol has poor water solubility and low bioavailability. Additionally, ergosterol has a Δ^5,7^ conjugated double bond system on its steroid nucleus B ring, which makes it chemically reactive and prone to photolysis under light (especially ultraviolet light) [18,19]. Under UV light, ergosterol readily undergoes photooxidation, forming vitamin D_2_ [9,26,27,28,29]. Ergosterol also exhibits some antioxidant properties. Under the influence of peroxide-free radicals, it can be oxidized to products such as ergosterol peroxide after prolonged exposure to air [9,29]. The structure of ergosterol peroxide is shown in Figure 1B. Moreover, ergosterol is thermally unstable. Its degradation follows first-order reaction kinetics, and its degradation rate increases significantly with temperature [26]. To overcome the low bioavailability of ergosterol due to its physicochemical properties, biotransformation or chemical structure modification techniques can be employed to improve its solubility and stability. Enzyme-catalysed systems or microbial transformations, along with studies based on structure–activity relationships, can modify specific sites or spatial structures of the ergosterol molecule, thereby altering its physicochemical properties. However, it is important to avoid altering the pharmacophore, as this may affect its efficacy. Alternatively, the bioavailability of ergosterol can be improved by delivery systems to enhance its absorption.

### 3.2. Biosynthesis, Natural Distribution, and Source Relevance

Ergosterol is mainly produced by fungi and is also present in some photosynthetic eukaryotes, particularly green algae. In most fungi, ergosterol is synthesized from lanosterol through the Δ24(28)-alkene pathway, whereas some algae use a cycloartenol-derived Δ25(27)-alkene pathway that resembles sterol biosynthesis in higher plants [27,28,30]. This pathway divergence is relevant not only for taxonomy but also for source selection, industrial production, and interpretation of ergosterol-related bioactivity, because sterol composition may vary substantially across species, developmental stages, culture conditions, and extraction procedures.

Although ergosterol is generally considered a major membrane sterol in Ascomycetes and Basidiomycetes, it is not universally distributed across all fungal lineages. Some early-diverging fungi and selected later-branching taxa contain little or no ergosterol, or replace it with other sterols [26]. Therefore, the term “fungal sterol” should not be interpreted as chemically uniform across all fungal sources. For pharmacological and formulation studies, this distinction is important because crude extracts or sterol-enriched fractions may contain parent ergosterol together with ergosterol peroxide, 22,23-dihydroergosterol, brassicasterol-related metabolites, and other fungal sterols. Such compositional heterogeneity may affect biological activity, pharmacokinetic interpretation, and reproducibility.

From a translational perspective, biosynthetic and source-related studies provide a foundation for improving ergosterol supply and developing ergosterol-derived products. Ergosterol biosynthesis can be affected by exogenous inducers, nutritional stress, ERG-gene regulation, homologous genes related to sterol synthesis and fungal development, and pathway engineering [31,32,33,34,35,36]. For example, methyl jasmonate can enhance ergosterol biosynthesis in fungal systems [31], whereas iron availability can regulate ERG-gene expression and sterol-pathway adaptation in yeast [32,33]. In addition, ERG4-related regulation and enzyme engineering of lanosterol 14α-demethylase may support strain improvement or yield optimization [34,35,36]. However, these strategies should be linked to downstream quality control, active-entity attribution, and formulation requirements. Future studies should report the biological source, extraction or production method, sterol composition, purity, and stability of ergosterol-containing materials, particularly when comparing disease-model efficacy or pharmacokinetic behavior across studies.

### 3.3. Ergosterol as a Fungal Membrane Sterol

**Figure 2:**
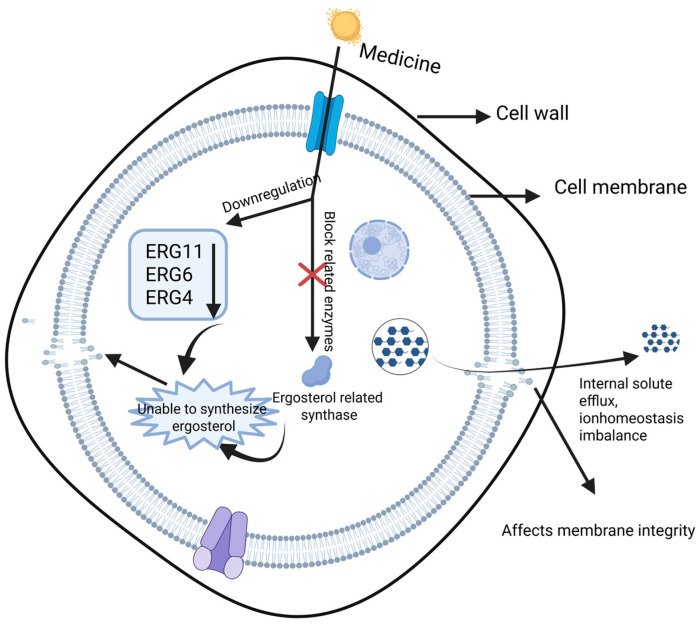
Inhibition of ergosterol synthesis leading to disruption of the cell membrane. Created in BioRender. Bai, D. (2026) https://BioRender.com/mxeq1af (accessed on 5 May 2026).

Ergosterol is a fungus-specific sterol and a major structural component of fungal cell membranes, playing a role analogous to cholesterol in mammalian membranes. It contributes to membrane integrity, fluidity, permeability, and the proper function of membrane proteins. Consequently, disrupting ergosterol biosynthesis or its membrane distribution can compromise membrane structure and homeostasis, ultimately leading to fungal cell death. Multiple antifungal agents exert activity by reducing cellular ergosterol content. For example, cinnamaldehyde, eugenol, and terbinafine can inhibit ergosterol production, resulting in membrane damage with increased permeability, potassium ion leakage, and loss of intracellular solutes, accompanied by changes in extracellular conductivity and disruption of ion gradients [37,38,39]. As shown in Figure 2, antimicrobial compounds may downregulate genes involved in ergosterol biosynthesis or inhibit key enzymes in the biosynthetic pathway, thereby decreasing ergosterol levels and destabilizing the membrane. This mechanism has been applied not only in clinical antifungal therapy but also in food preservation settings, such as inhibiting *Aspergillus flavus* on grain products [40].

At the molecular level, several natural products and bioactive compounds reduce ergosterol by suppressing ERG genes (e.g., ERG11, ERG6, and ERG4), leading to membrane rupture and antifungal effects [41,42,43]. Clinically, azoles inhibit lanosterol 14α-demethylase, while tolnaftate targets squalene epoxidase, thereby blocking ergosterol biosynthesis and exerting fungistatic or fungicidal activity [44]. Similar sterol-targeting concepts have also been explored against parasites such as *Leishmania* [45]. In addition to synthesis inhibition, altered sterol trafficking can attenuate fungal virulence. For instance, deletion of Ysp2 leads to abnormal accumulation of ergosterol in the plasma membrane, membrane invagination, and cell wall defects, thereby reducing the virulence of *Cryptococcus neoformans* [46]. Moreover, ergosterol photosensitivity has been exploited in photodynamic strategies that generate reactive oxygen species and oxidized sterols (e.g., peroxides), which can damage fungal membranes [47].

However, resistance driven by adaptive sterol homeostasis and pathway mutations remains a key limitation of sterol-targeting antifungals; therefore, inhibitors targeting distinct targets within the ergosterol pathway, together with systematic resistance profiling, are important for improving long-term efficacy [48].

### 3.4. Ergosterol as a Precursor of Vitamin D_2_

Ergosterol is an important precursor of vitamin D_2_ and represents a major non-animal source for vitamin D supplementation. Under ultraviolet irradiation, ergosterol undergoes photochemical conversion to vitamin D_2_ [49,50]. This conversion can occur across a broad UV range (290–400 nm) [50,51,52,53], while higher conversion efficiency has been reported within 290–315 nm, which is commonly used in industrial production and most experimental studies [50,51,53]. Accordingly, UV treatment has been widely applied to increase vitamin D_2_ content in foods, especially mushrooms [50,51,52].

Beyond conventional irradiation, process and structural engineering approaches have been explored to improve conversion efficiency. For example, 4D printing of purple sweet potato paste containing ergosterol was reported to increase the effective exposure area under UV and enhance conversion efficiency, enabling the production of vitamin D_2_-enriched foods from lower-cost raw materials [49,54]. In addition, food processing and storage conditions can influence vitamin D_2_ retention and the stability of intermediates, thereby affecting final product quality [50,51,52,53].

Nevertheless, reported yields vary substantially across studies due to differences in irradiation wavelength/dose, exposure time, sample geometry, and matrix composition; therefore, standardized reporting of UV parameters and quantification of conversion efficiency (including by-product profiling) are important for comparability and safety evaluation.

### 3.5. Ergosterol as an Analytical Marker of Fungal Biomass

Ergosterol is a sterol largely restricted to fungi and, therefore, has long been used as a practical chemical marker for estimating fungal contamination or fungal biomass in complex matrices such as foods, agricultural products, environmental samples, and indoor materials [55,56,57,58]. This proxy has also enabled analytical workflows to complement culture-based enumeration, particularly in matrices where microbial cells are difficult to isolate [55,56,57].

In food and agriculture, ergosterol quantification has been used as an index of fungal presence in grain-related matrices, with chromatographic detection (typically HPLC-based) [55]. In studies examining mold contamination and mycotoxin risk, ergosterol-linked measurements have been assessed alongside ochratoxin A, supporting the concept that ergosterol can contribute to contamination evaluation and risk monitoring, while acknowledging that the relationship can depend on commodity and contamination context [59]. For liquid foods, ergosterol has also been measured in juices using rapid spectrophotometric approaches and proposed as a faster alternative for estimating spoilage-related fungal load under defined conditions [60]. In environmental and built environments, ergosterol has been widely used to reflect fungal biomass in soils and contaminated settings. It has been applied in studies involving pollutants such as heavy metals and pesticides/fungicides, supporting its usefulness for comparing fungal biomass patterns across treatments [56,57]. Ergosterol has also been used in damp building materials to evaluate mold growth and to provide an approximate indication of contamination severity across locations within a material [58]. In a clinical-oriented analytical development, an LC–MS/MS method was developed to quantify ergosterol in cerebrospinal fluid (CSF); ergosterol was not detected in CSF samples inoculated with bacteria, supporting its potential utility for differentiating bacterial from non-bacterial etiologies in proof-of-concept settings [61].

Future work should prioritize method harmonization across matrices (sample preparation, recovery, stability, and reporting of detection limits) to improve cross-study comparability [55,56,57]. In application-focused settings, defining context-specific thresholds (for example, what ergosterol level corresponds to actionable spoilage or indoor-contamination risk in a given matrix) would improve decision-making utility [58,60]. In clinical contexts, ergosterol detection may be most defensible as a rule-in signal for non-bacterial processes rather than a species-specific diagnosis, and additional validation is required before routine deployment [61].

## 4. Pharmacokinetic Limitations and ADME Barriers

Pharmacokinetic limitations are central to evaluating the translational potential of ergosterol. Although ergosterol and its derivatives have been associated with diverse biological activities, it remains uncertain whether many of these effects can be achieved at pharmacologically relevant systemic or tissue exposure levels. Free ergosterol exhibits limited oral bioavailability, largely because of its low aqueous solubility, limited micellar bioaccessibility, incomplete intestinal absorption, metabolic conversion, and fecal elimination [10,11,12,62,63,64]. During digestion, fungal sterols such as ergosterol can compete with cholesterol for incorporation into dietary mixed micelles (DMMs) because of their structural similarity; however, coexisting fungal β-glucans may also scavenge fungal sterols or reduce their micellar availability, thereby limiting the fraction available for absorption [62]. In classical radiolabeled studies using orally administered ergosterol-C14 in rats, only approximately 2–5% of the administered dose was absorbed, mainly via the lymphatic system, whereas most radioactivity remained in the gastrointestinal tract and feces [63]. LC–MS-based pharmacokinetic studies have further shown that ergosterol can be detected in rat plasma, urine, and feces after oral administration, with fecal recovery representing a major elimination route [12]. Stable isotope-tracing studies further demonstrated that, after oral administration of ergosterol-d1, the peak serum concentration of brassicasterol-d1 was higher than that of ergosterol-d1, indicating that metabolic conversion may substantially influence systemic exposure profiles [64]. Therefore, this section discusses the major ADME barriers of ergosterol, with emphasis on intestinal bioaccessibility, tissue exposure, parent compound–metabolite conversion, excretion, and their implications for exposure–response evaluation.

### 4.1. Absorption and Intestinal Bioaccessibility

The first pharmacokinetic barrier for ergosterol is its poor intestinal bioaccessibility. Due to its low polarity and poor aqueous solubility, ergosterol depends largely on micellar solubilization during digestion before it can become available for intestinal uptake [10,62]. Classical radiolabeled studies using orally administered ergosterol-C14 in rats showed that only approximately 2–5% of the administered dose was absorbed, mainly via the lymphatic system, whereas most of the radioactivity remained in the gastrointestinal tract and feces [63]. This finding indicates that the oral absorption of free ergosterol is intrinsically limited.

Because of its structural similarity to cholesterol, ergosterol and other fungal sterols can compete with cholesterol for incorporation into DMMs, a mechanism mainly discussed in relation to reduced intestinal cholesterol absorption [62]. Coexisting fungal β-glucans may further modify this process by promoting cholesterol displacement from DMMs while partially scavenging fungal sterols, thereby reducing the micellar availability of ergosterol [62]. The intestinal uptake of sterol-like molecules may involve transport-related pathways relevant to cholesterol or vitamin D absorption, including Npc1l1, Cd36, Scarb1, Abca1, and Abcg5/8; however, direct transporter involvement in ergosterol absorption remains insufficiently established [10]. In mice, dietary ergosterol did not significantly alter the mRNA abundance of several intestinal sterol transporters, indicating that the relationship between ergosterol, micelle formation, transporter expression, and intestinal uptake requires further investigation [10]. In a rat pharmacokinetic study, a single oral dose of 100 mg/kg ergosterol resulted in measurable concentrations of ergosterol in plasma, urine, and fecal samples over 36 h, indicating that a fraction of orally administered ergosterol can enter systemic circulation [12]. A stable isotope-labeled study using orally administered ergosterol-d1 also demonstrated measurable serum ergosterol-d1 concentrations after dosing [64]. Ergosterol peroxide, a distinct oxidized derivative, showed acceptable tolerability in mice, with a reported maximum tolerated dose of 500 mg/kg; however, this result should be interpreted as specific to ergosterol peroxide rather than as direct pharmacokinetic evidence for parent ergosterol [65].

Overall, the magnitude of ergosterol absorption appears to vary with model, dose, food matrix, formulation, and compound form. Direct human absorption data remain limited; therefore, quantitative conclusions should be interpreted within the specific experimental context of each study.

### 4.2. Distribution and Tissue Exposure

Available evidence suggests that ergosterol or ergosterol-derived sterols can reach systemic and tissue compartments under certain experimental conditions, but the interpretation of tissue exposure is affected by dose, model, route of administration, and metabolic conversion. Early ergosterol-C14 studies showed that, after oral administration in rats, a small absorbed fraction localized mainly in the liver, lung, spleen, and adrenal glands during the early observation period [63]. In the same study, intravenous administration resulted in higher early hepatic radioactivity, with additional recovery in the lung, spleen, adrenal glands, and gastrointestinal contents over time, suggesting that hepatic handling and excretory redistribution are important components of ergosterol disposition [63].

Dietary studies also indicate that systemic parent-compound exposure may occur under specific dosing conditions. In mice, higher dietary ergosterol exposure produced detectable serum ergosterol concentrations, whereas lower-dose exposure resulted in serum concentrations below the limit of quantification [10]. In rats receiving orally administered stable isotope-labeled ergosterol-d1, serum concentration–time profiles of ergosterol-d1 were also obtained [64]. By contrast, in ovariectomized rats fed a high-ergosterol diet, plasma ergosterol was below the limit of quantification, whereas brassicasterol was detectable, supporting substantial conversion of absorbed ergosterol to this metabolite [11]. Stable isotope-tracing studies also showed that the peak serum concentration of brassicasterol-d1 was higher than that of ergosterol-d1 after oral ergosterol-d1 administration [64]. These findings indicate that systemic or tissue exposure should not be interpreted by treating all ergosterol-related sterols as a single entity.

Parent ergosterol, brassicasterol, ergosterol peroxide, glycosylated metabolites, hydroxylated metabolites, and other oxidized derivatives may differ in tissue distribution, persistence, biological activity, and safety profile. Systematic tissue distribution studies remain limited, particularly with respect to time-course profiles, quantitative parent/metabolite separation, target-tissue exposure, and route-dependent disposition. This limitation makes it difficult to directly connect reported biological activities, such as anticancer, neuroprotective, or metabolic effects, with pharmacologically plausible tissue concentrations.

### 4.3. Metabolism and Parent Compound–Metabolite Conversion

Metabolism represents a key barrier to assigning biological activity to parent ergosterol. Available evidence indicates that ergosterol can intersect with cholesterol- and vitamin D-related sterol pathways. A major reported metabolic route involves 7-dehydrocholesterol reductase (DHCR7), which catalyzes the conversion of ergosterol to brassicasterol. In rat studies, absorbed ergosterol was reported to be rapidly metabolized to brassicasterol within approximately 25 h after ingestion [11]. This conversion is important because biological effects observed after oral ergosterol administration may reflect the activity of brassicasterol or other metabolites rather than the parent compound itself.

LC–MS-based pharmacokinetic analysis in rats identified two ergosterol-related metabolites, ERG1 and ERG2, in plasma after oral administration, indicating that orally administered ergosterol undergoes metabolic transformation rather than remaining solely as unchanged parent compound [12]. Stable isotope-tracing studies further enabled the simultaneous quantification of serum ergosterol-d1 and brassicasterol-d1 and showed that the peak serum concentration of brassicasterol-d1 was higher than that of ergosterol-d1 after oral ergosterol-d1 administration [64]. Earlier radiolabeling studies also showed that, after intravenous administration of ergosterol-C14, none of the radioactivity recovered in bile could be identified as unchanged ergosterol, indicating metabolic conversion before biliary elimination [63].

In addition to DHCR7-mediated conversion and glycosylated metabolite formation, ergosterol can undergo hydroxylation and oxidative metabolism. Cytochrome P450scc has been shown to metabolize ergosterol in a reconstituted enzymatic system and in isolated adrenal mitochondria, generating 17α,24-dihydroxyergosterol as the major product and hydroxyergosterol as a minor intermediate; importantly, side-chain cleavage was not observed under those conditions [66]. Earlier mitochondrial studies demonstrated that rat and mouse liver mitochondria can oxidize radiolabeled ergosterol, yielding radioactive CO_2_ and acidic radioactive products [67]. Ergosterol-containing extracts have also been reported to modulate sterol metabolism-related gene expression in cell and animal models, including LDLR/HMGCR-related pathways; however, attribution of these effects to ergosterol itself should consider extract composition, dose, model, and experimental design [10,62].

Overall, existing studies support multiple metabolic routes, including DHCR7-mediated conversion to brassicasterol, LC–MS-detected glycosylated metabolites, stable isotope-tracing-confirmed brassicasterol formation, P450scc-mediated hydroxylation, and mitochondrial oxidation. However, the relative contribution of these pathways *in vivo* has not been quantitatively resolved. Future studies should therefore include metabolite profiling, parent/metabolite-specific quantification, and pathway validation before attributing downstream biological effects to ergosterol itself.

### 4.4. Excretion and Mass-Balance Limitations

Ergosterol-related sterols are eliminated predominantly through the fecal route, consistent with limited intestinal absorption and low oral bioavailability. In a rat pharmacokinetic study using RRLC–MS/MS, ergosterol was detected in plasma, urine, and fecal samples after a single oral dose of 100 mg/kg; fecal levels were higher than plasma and urinary levels, and approximately 62.5% of the administered dose was cumulatively recovered in feces [12]. Classical ergosterol-C14 studies similarly showed that most orally administered radioactivity remained in the gastrointestinal tract and feces, whereas urinary recovery was minimal [63]. These findings indicate that fecal elimination is a major route for orally administered free ergosterol.

Fecal recovery may vary depending on dose, formulation, food matrix, and coexisting matrix components. For example, β-glucan-containing extracts may alter the micellar behavior of fungal sterols during digestion, thereby affecting intestinal bioaccessibility and fecal elimination [62]. In addition to fecal elimination, earlier radiolabeling studies suggested that bile represents an important excretory pathway for ergosterol-derived metabolites, although unchanged ergosterol was not identified in bile [63]. Mitochondrial oxidation studies also demonstrated the formation of radioactive CO_2_ and acidic radioactive products from radiolabeled ergosterol, suggesting that metabolic elimination routes may contribute to ergosterol disposition [67].

A major limitation of the current literature is the lack of complete mass-balance studies using modern parent/metabolite-resolved analytical methods. Many reports do not clearly distinguish unchanged parent ergosterol from brassicasterol, glycosylated metabolites, hydroxylated metabolites, oxidized derivatives, or other sterol-related products in feces, bile, urine, plasma, or tissues. As a result, the relative contributions of non-absorption, intestinal metabolism, hepatic metabolism, biliary elimination, urinary excretion, and fecal excretion remain incompletely defined. Validated LC–MS/MS methods and stable isotope-tracing strategies should be extended from serum analysis to feces, urine, bile, and tissue samples to enable more comprehensive mass-balance evaluation [64]. To further quantify these ADME limitations and evaluate whether formulation strategies can improve *in vivo* exposure, the available *in vivo* pharmacokinetic and exposure data are summarized in Table 1. This table includes studies directly related to *in vivo* pharmacokinetics or exposure, including free ergosterol, radiolabeled ergosterol, stable isotope-labeled ergosterol, and formulations with plasma concentration or tissue distribution data.

**Figure 3:**
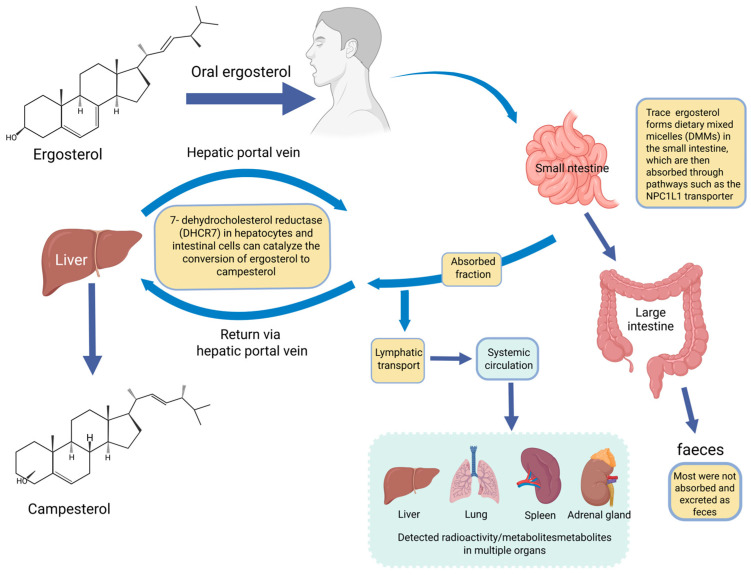
Major absorption and metabolic pathways of ergosterol. Created in BioRender. Bai, D. (2026) https://BioRender.com/jz3ycxg (accessed on 5 May 2026).

The major absorption and metabolic pathways of ergosterol are summarized in Figure 3.

### 4.5. Pharmacokinetic Implications for Translational Development

The pharmacokinetic profile of ergosterol has direct implications for its translational development. Biological activities reported *in vitro* or in animal models should be interpreted in relation to achievable systemic and tissue exposure; otherwise, it remains difficult to determine whether the proposed mechanisms are pharmacologically plausible under physiologically or clinically relevant conditions. Current evidence indicates that free ergosterol has limited oral bioavailability, largely due to poor aqueous solubility, limited intestinal bioaccessibility, fecal elimination, and metabolic conversion. Even when absorbed, ergosterol may undergo conversion to brassicasterol via DHCR7, resulting in low or undetectable circulating levels of the parent compound under some conditions [11]. Stable isotope-labeled studies further showed that the peak serum concentration of brassicasterol-d1 was higher than that of ergosterol-d1 after oral ergosterol-d1 administration, indicating that metabolite exposure may exceed parent-compound exposure [11].

Future pharmacokinetic studies should therefore prioritize validated analytical methods for the separate quantification of parent ergosterol, brassicasterol, ergosterol peroxide, glycosylated metabolites, hydroxylated metabolites, and other oxidized derivatives. Formulation studies should also move beyond apparent bioavailability enhancement and report Cmax, Tmax, AUC, half-life, tissue distribution, metabolite profiles, excretion routes, and safety parameters. Integrating stable isotope tracing, mass-balance assessment, tissue distribution analysis, and pharmacokinetic–pharmacodynamic modeling will be essential for determining whether ergosterol-based compounds are more suitable as orally active agents, formulation-enhanced therapeutics, functional ingredients, or scaffolds for derivative design.

## 5. Active Entities: Parent Ergosterol, Ergosterol Peroxide, and Metabolites

Ergosterol-related studies involve several chemically and pharmacokinetically distinct entities, including parent ergosterol, ergosterol peroxide, brassicasterol, glycosylated metabolites, hydroxylated metabolites, and other oxidized products. These entities differ in oxidation state, systemic exposure, metabolic stability, and dominant pharmacological profiles. Clarifying the investigated entity provides the basis for evaluating dose–exposure relationships, mechanistic plausibility, and translational development strategies in the disease-oriented sections below.

### 5.1. Rationale for Active-Entity Distinction

In ergosterol research, the administered compound and the entity that mediates the biological effect are not always identical. In *in vitro* experiments, activity can often be attributed more directly to the compound added to the system, such as ergosterol or ergosterol peroxide. In contrast, after oral administration, ergosterol may undergo intestinal absorption, hepatic handling, enzymatic conversion, and oxidative metabolism, generating brassicasterol or other ergosterol-related metabolites. Pharmacokinetic studies have detected ergosterol-related metabolites after oral ergosterol exposure, and stable isotope-labeled studies further showed that the peak serum concentration of brassicasterol-d1 can exceed that of ergosterol-d1 after oral administration of ergosterol-d1 [11,12,64]. Therefore, biological effects after oral ergosterol exposure should be interpreted in relation to both parent-compound and metabolite exposure.

### 5.2. Parent Ergosterol

Parent ergosterol is a natural fungal sterol and is the main administered compound in many nutritional, metabolic, and organ-protective studies. Because of its structural similarity to cholesterol, it is closely related to mixed micelle formation, cholesterol absorption, sterol homeostasis, and membrane-associated processes. In disease models, parent ergosterol has been associated with glucose and lipid metabolism, cholesterol regulation, anti-inflammatory activity, antioxidant effects, renal protection, pulmonary protection, and selected anticancer effects. Across preclinical studies, parent ergosterol has been evaluated in metabolic, inflammatory, renal, hepatic, pulmonary, and neurological models [2,3,4,73,74,75,76,77,78]. Cancer-related evidence has also linked parent ergosterol to tumor growth [13,67,79], sterol homeostasis [15,80], and treatment-response models [81]. The molecular changes reported for parent ergosterol are context-dependent and include sterol-homeostasis regulation, inflammatory signaling, apoptosis-related proteins, and drug-efflux markers, with the level of target evidence varying across models. However, because orally administered ergosterol has limited absorption and can undergo metabolic conversion, effects attributed to parent ergosterol *in vivo* should be interpreted in relation to parent-compound exposure, administration route, and dose conditions.

### 5.3. Ergosterol Peroxide as an Oxidized Derivative

Ergosterol peroxide is a representative oxidized derivative in ergosterol-related research. Compared with parent ergosterol, EP contains an additional peroxide structure and shows a distinct bioactivity profile, especially in cancer-related models. Current studies are mainly concentrated in tumor models, where EP is more frequently associated with pro-apoptotic, anti-proliferative effects [82], anti-migratory and anti-invasive activity [83], ROS-related mitochondrial stress [84], autophagy-modulating effects [85], and radiosensitizing effects [86]. Reported mechanisms include β-catenin suppression [14], DR5/FADD/caspase-8/3 activation [82], PI3K/AKT/mTOR modulation [87], STAT3/NF-κB regulation [88], AKT/c-Myc/FOXO3-related apoptosis [17], and ROS-dependent cell death [85]. Selected non-cancer studies also suggest anti-inflammatory, antifibrotic, or neuronal-repair-related activities, but these findings remain more limited [89,90,91]. These features support discussing EP as a representative oxidized derivative within ergosterol-related bioactivity studies, particularly when evaluating cancer-related mechanisms.

### 5.4. Brassicasterol and Other Ergosterol-Related Metabolites

Brassicasterol is one of the most relevant metabolism-related entities after oral ergosterol exposure. Ergosterol can be converted to brassicasterol through a DHCR7-mediated route [65]. Stable isotope-labeled studies further showed that, after oral administration of ergosterol-d1, the peak serum concentration of brassicasterol-d1 was higher than that of ergosterol-d1, indicating that brassicasterol can become an important systemic exposure entity after oral ergosterol administration [64]. In disease models, the relevance of brassicasterol is particularly evident in bladder carcinogenesis studies, where the inhibitory effect associated with oral ergosterol exposure has been linked to brassicasterol-mediated modulation of androgen signaling [92,93].

In addition to brassicasterol, pharmacokinetic studies have identified other ergosterol-related metabolites, including ERG1 and ERG2 [12]. Ergosterol can also undergo P450scc-mediated hydroxylation to generate hydroxylated products such as 17α,24-dihydroxyergosterol and can form CO_2_ and acidic oxidation products in hepatic mitochondrial systems [66,67]. These studies indicate that ergosterol does not remain solely as an unchanged parent compound. Although the specific pharmacological roles of many metabolites remain insufficiently defined, they are important for interpreting *in vivo* effects after oral ergosterol exposure and for guiding translational development.

### 5.5. Implications for Interpreting Disease-Oriented Evidence

Together, these active entities provide different interpretative frameworks for disease-oriented evidence. *In vitro* studies using purified compounds can generally be linked to the tested molecule, whereas oral administration studies require consideration of absorption, metabolism, and tissue exposure. For mixed extracts or multi-component formulations, the observed activity may reflect combined contributions from parent ergosterol, oxidized derivatives, metabolites, and matrix components. This framework is used below to organize disease-related evidence according to compound form, model context, and translational relevance.

## 6. Disease-Oriented Evidence for Ergosterol-Based Bioactivities

After distinguishing parent ergosterol, ergosterol peroxide, and ergosterol-related metabolites, the disease-oriented evidence can be further organized according to the pathological context. Current studies mainly focus on cancer, metabolic disorders, inflammation and organ injury, neuroprotection, gut microbiota-related disorders, and other emerging disease models. Most evidence is derived from cellular experiments and animal studies; therefore, these findings should be regarded as preclinical signals rather than established therapeutic conclusions. In the following subsections, disease-related effects are discussed in relation to the investigated entity, experimental model, mechanistic evidence, and translational limitations.

### 6.1. Anticancer Effects

Cancer is one of the most extensively investigated disease areas for ergosterol-related compounds. Current evidence involves parent ergosterol, ergosterol peroxide, ergosterol peroxide derivatives, and selected metabolites. Overall, parent ergosterol is more closely associated with FOXO3, AKT/GSK-3β/β-catenin, LXR/ABCA1, and multidrug-resistance-related regulation, whereas ergosterol peroxide is more frequently linked to ROS-associated mitochondrial injury, apoptosis, autophagy, β-catenin suppression, death-receptor signaling, and radiosensitization. Therefore, anticancer evidence should be interpreted according to compound type and tumor context.

Parent ergosterol has shown anticancer or tumor-modulating effects in breast cancer, colorectal cancer, bladder carcinogenesis, and multidrug-resistant tumor models. In breast cancer models, ergosterol inhibited proliferation and malignant phenotypes in MCF-7 and MDA-MB-231 cells, accompanied by suppression of AKT/GSK-3β/β-catenin-related signaling readouts, and promoted apoptosis-related changes involving BAX, caspase-7, PARP, BCL-2, and STAT3 [13,79]. Ergosterol purified from *Amauroderma rude* upregulated FOXO3 and downstream apoptotic mediators such as Fas, FasL, BimL, and BimS, and prolonged survival in a murine tumor challenge model [1]. In colorectal cancer models, ergosterol and related sterols showed LXR-related activity and altered cholesterol-homeostasis targets, including ABCA1, ABCG1, and ApoE [15,80]. In bladder carcinogenesis models, ergosterol suppressed tumor-promotion-related molecules, including cyclin D1, COX-2, 5α-reductase type 2, and androgen receptor, and this effect may be associated with brassicasterol-mediated modulation of androgen signaling [92,93]. In multidrug-resistant gastric cancer cells, ergosterol increased intracellular accumulation of adriamycin and Rh123, inhibited MDR1 transcription, downregulated P-gp expression, and enhanced adriamycin sensitivity [81].

Ergosterol peroxide shows a stronger association with cytotoxic, pro-apoptotic, and anti-invasive activity in tumor models. In breast cancer models, ergosterol peroxide and its derivatives have been linked to inhibition of migration, invasion, and metastasis, mitochondrial dysfunction, ROS accumulation, and PI3K/AKT/mTOR-related protein changes [83,84,87]. In colorectal cancer, ergosterol peroxide suppressed β-catenin and downstream targets such as c-Myc, cyclin D1, and CDK-8, induced apoptosis, and reduced tumor burden [14,94]. In prostate cancer models, EP inhibited prostate cancer cell growth and induced apoptosis, with evidence of caspase-3 activation and DNA fragmentation [95], and DR5/FADD/caspase-8/3 signaling was further implicated in DU145 cells [82]. In ovarian cancer models, EP was associated with β-catenin downregulation and SHP2/Src–STAT3-related changes [88]. In hepatocellular carcinoma and lung cancer models, EP-related effects involved AKT/c-Myc/FOXO3-associated apoptosis [17] and ROS-dependent apoptosis and autophagy [85], respectively. In cervical cancer models, EP showed radiosensitizing activity [86].

**Figure 4:**
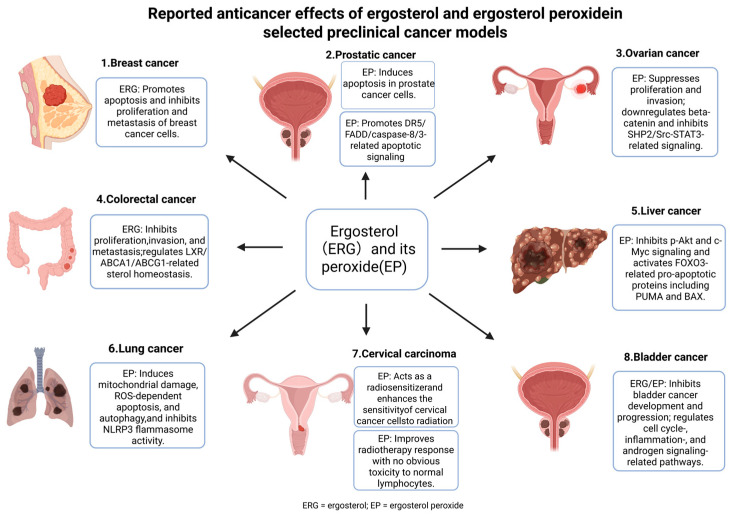
Effects of ergosterol and ergosterol peroxide on selected cancers. Created in BioRender. Bai, D. (2026) https://BioRender.com/uo3qm3s (accessed on 5 May 2026).

Overall, both parent ergosterol and ergosterol peroxide show anticancer potential, but their mechanistic emphasis differs. Parent ergosterol is more closely related to tumor-associated metabolism, sterol homeostasis, FOXO3/β-catenin signaling, and drug-resistance modulation, whereas ergosterol peroxide is more strongly associated with ROS stress, mitochondrial injury, apoptosis, autophagy, and radiosensitization. The major anticancer mechanisms and representative cancer types are summarized in Figure 4 and Table 2. Most evidence remains preclinical, and systematic pharmacokinetic profiling, tumor-site exposure data, and dose–exposure–response validation are still limited. Future anticancer studies should clearly distinguish parent ergosterol, ergosterol peroxide, derivatives, and related metabolites while integrating formulation strategies to improve tumor-site exposure and *in vivo* validation.

### 6.2. Metabolic Regulation: Glucose, Lipids, Cholesterol, and Uric Acid

The metabolic effects of ergosterol are mainly related to glucose, lipid, cholesterol, and uric acid homeostasis. These topics are connected by intestinal sterol absorption, mixed micelle formation, hepatic metabolism, renal transport, inflammation, and oxidative stress. Unlike the anticancer field, the evidence for metabolic regulation is primarily centered on parent ergosterol or ergosterol-containing interventions rather than ergosterol peroxide.

In diabetes and diabetic nephropathy models, ergosterol has been reported to improve glucose handling, insulin resistance, dyslipidemia, inflammation, and renal injury. In L6 myotubes and KK-Ay mice with spontaneous type 2 diabetes, ergosterol enhanced glucose uptake, promoted GLUT4 translocation and expression, and improved insulin resistance, accompanied by changes in Akt- and PKC-related phosphorylation readouts [3]. In STZ-induced diabetic mice, ergosterol reduced blood glucose, uric acid, creatinine, TG, and TC, increased serum insulin, and alleviated renal pathological injury, with associated changes in PI3K/Akt/NF-κB-related protein readouts [73]. In an STZ-induced diabetic nephropathy model, ergosterol reduced renal inflammatory cytokines, including IL-6, TNF-α, and MCP-1, and decreased renal p-NF-κB, COX-2, and iNOS expression [74]. In db/db mice and high-glucose-induced HK-2 cells, ergosterol restored CPT1A expression and was linked to FOXA1/CPT1A-associated fatty acid oxidation, thereby reducing renal lipid accumulation and inflammatory macrophage polarization [75]. In STZ-induced diabetic mice and high-glucose-induced mesangial cells, ergosterol reduced mesangial cell proliferation and ECM deposition, accompanied by reduced TGF-β1/Smad2-related profibrotic signaling and changes in MMP-2 and MMP-9 [76].

For cholesterol regulation, ergosterol mainly acts through intestinal sterol handling and hepatic cholesterol metabolism. Due to its structural similarity to cholesterol, ergosterol can compete for incorporation into dietary mixed micelles, reduce cholesterol uptake, and promote fecal cholesterol excretion [4,62,77]. At the hepatic and systemic levels, ergosterol has been reported to alter cholesterol metabolism-related readouts, including SREBP-2, LDL-R, HMG-CoR, CYP7A1, and LXR-α in high-cholesterol diet-fed rats, while ergosterol-enriched extracts affected LDLR and intestinal Srebf2/Nr1h4-related transcriptional responses in cell and mouse models [4,62]. For uric acid regulation, ergosterol-related interventions have been reported to reduce uric acid levels in hyperuricemia or gouty nephropathy models. The available evidence includes enzyme-level inhibition of XO and COX-2 as well as model-based changes involving URAT1, GLUT9, ABCG2, NLRP3, and IL-1β-related readouts [5,96].

Together, these findings place ergosterol-related metabolic effects at the intersection of glucose uptake, fatty acid oxidation, cholesterol absorption and metabolism, uric acid production, urate transport, inflammation, and kidney protection. Mixed-micelle competition, DHCR7-mediated conversion, FOXA1/CPT1A-associated fatty acid oxidation, and XO/COX-2 inhibition have relatively stronger mechanistic support, whereas PI3K/Akt, NF-κB, TGF-β1/Smad2, and urate-transporter changes are best described as pathway- or protein-level readouts within specific disease models. Future studies should connect metabolic endpoints with *in vivo* exposure, dose–exposure–response relationships, and formulation optimization.

### 6.3. Anti-Inflammatory and Organ-Protective Effects

Ergosterol and related compounds have been investigated for anti-inflammatory and organ-protective effects in models of acute lung injury, chronic obstructive pulmonary disease, skin inflammation, hepatic fibrosis, renal fibrosis, and drug-induced kidney injury. A common feature across these studies is the modulation of inflammatory mediators, oxidative-stress markers, fibrosis-associated proteins, and tissue-injury readouts. Compared with ergosterol peroxide in tumor models, parent ergosterol in organ-protective settings is generally associated with anti-inflammatory, antioxidant, or antifibrotic effects.

In an LPS-induced acute lung injury model, ergosterol pretreatment reduced pulmonary pathological damage and edema, decreased pro-inflammatory cytokines such as TNF-α and IL-6, and lowered NF-κB-, COX-2-, and iNOS-related inflammatory readouts [2]. In a cigarette smoke-induced COPD mouse model, intragastric administration of ergosterol improved oxidative stress-related readouts, including increased SOD and CAT and decreased MDA, while reducing inflammatory cytokines such as TNF-α, IL-6, and IL-1β in serum and lung tissue. These effects were accompanied by reduced JAK3/STAT3/NF-κB-related inflammatory signaling [97]. These findings suggest that ergosterol acts mainly as an anti-inflammatory and antioxidant modulator in inflammatory lung injury.

Beyond pulmonary models, ergosterol has been reported to affect skin inflammation, hepatic fibrosis, renal fibrosis, and drug-induced kidney injury. In LPS- and UVA-induced HaCaT keratinocyte inflammatory models, ergosterol and ergosterol peroxide reduced ROS, IL-8, and IL-1β, promoted AQP3 and FLG expression, and decreased PI3K/Akt-related gene-expression readouts [90]. In renal fibrosis-related cellular models, ergosterol peroxide attenuated TGF-β1-induced NRK-49F fibroblast activation, ECM production, and MAPK phosphorylation readouts involving ERK1/2, p38, and JNK [91]. In AGE-induced HSC-T6 hepatic stellate cells, ergosterol reduced α-SMA, MMP-9, EMT-related markers, and AGE/RAGE-associated activation. This antifibrotic response was supported by PPARγ nuclear translocation, PPARγ reporter activity, and attenuation of ergosterol effects after PPARγ knockdown, indicating PPARγ-dependent pathway involvement [98]. Ergosterol also showed protective activity in gentamicin-induced MDCK cell injury by reducing oxidative stress, inflammatory markers, apoptosis-related readouts, and autophagy dysregulation [7]. Overall, these studies suggest that ergosterol and related derivatives may modulate inflammatory and tissue-injury processes, but the evidence is distributed across different organs and experimental models. More *in vivo* exposure data and causal mechanistic validation are required.

### 6.4. Neuroprotection and Neuronal Repair

Neuroprotective evidence is mainly derived from animal and cellular models involving neuroinflammation, oxidative stress, and neurite outgrowth. In LPS- or bisphenol A-induced neuroinflammation models, ergosterol reduced microglial activation and pro-inflammatory cytokine production, accompanied by NF-κB-, AKT-, and MAPK-related inflammatory readouts [16,99]. In a TNF-α-induced model of hippocampal neuronal injury, ergosterol increased Akt-related phosphorylation readouts, enhanced antioxidant defense such as SOD-1 activity, facilitated ROS clearance, and mitigated oxidative stress-related neuronal damage [6]. Ergosterol has also been reported to alter neurotransmission-related markers, including EGR1 and Grin2b, which may contribute to maintaining neuronal excitability under inflammatory stress [6]. These findings suggest a relationship with antioxidant and neurotransmission-related responses, but they do not establish Akt or NMDA receptor subunits as direct binding targets of ergosterol.

In addition to parent ergosterol, selected ergosterol derivatives, particularly ergosterol peroxide, have been reported to stimulate NGF-mediated neurite outgrowth in PC12 cells, suggesting possible relevance to neuronal repair and regeneration [89]. However, current evidence remains mainly animal- or cell-based, and data on brain exposure, blood–brain barrier penetration, and dose–exposure–response relationships are limited. Therefore, the neuroprotective effects of ergosterol-based compounds should be regarded as promising preclinical signals rather than established neurological therapeutic effects. The major lung- and nervous-system-related protective effects, experimental models, pathway readouts, and outcomes are summarized in Table 3.

### 6.5. Other Emerging Disease-Related Activities

Beyond cancer, metabolic disease, inflammatory organ injury, and neuroprotection, ergosterol has been associated with gut microbiota modulation, intestinal barrier function, irritable bowel syndrome, obesity-related cognitive impairment, osteoarthritis, and other tissue-protective effects. In a high-fat diet-induced obesity and cognitive impairment model, ergosterol administration regulated gut microbiota composition, improved lipid and oxidative-stress-related readouts, and reduced inflammation-related markers, suggesting possible involvement of gut–metabolic–brain interactions in metabolic and neurobehavioral outcomes [100]. In irritable bowel syndrome-related studies, ergosterol improved visceral hypersensitivity, intestinal motility, colonic inflammation, barrier integrity, and mast cell activation. Mechanistically, ergosterol reshaped gut microbiota, enhanced tryptophan metabolism, increased the microbiota-derived metabolite indole-3-lactate, and activated colonic AhR signaling; antibiotic treatment abolished the beneficial effects of ergosterol, and indole-3-lactate supplementation reproduced several effects on motility, hypersensitivity, and AhR-related readouts [101]. This evidence supports a microbiota–metabolite–AhR axis rather than direct AhR liganding by ergosterol.

In osteoarthritis models, ergosterol delayed cartilage degeneration and affected processes related to inflammation and extracellular matrix degradation. Mechanistic evidence involved increased Nrf2 and HO-1 expression, Nrf2 nuclear translocation, and HO-1 promoter luciferase activity, supporting Nrf2/HO-1 pathway activation in chondrocytes and cartilage [102]. However, direct binding of ergosterol to Nrf2 or its regulatory proteins has not been established. These findings remain preclinical and are best viewed as disease-context-specific signals rather than established therapeutic effects. These emerging directions broaden the potential application scope of ergosterol-based compounds, but most remain at an early exploratory stage. Future studies are needed to clarify the specific active entity involved, dose rationale, *in vivo* exposure, disease-relevant endpoints, and long-term safety.

### 6.6. Evidence Strength and Translational Limitations

Overall, disease-oriented evidence for ergosterol-related compounds is broad but uneven. Anticancer and metabolic regulation studies are relatively abundant and mechanistically diverse, whereas lung injury, neuroprotection, gut microbiota modulation, osteoarthritis, fibrosis, and other organ-protective effects remain at earlier exploratory stages. Most studies rely on *in vitro* assays or animal models, and human pharmacokinetic, clinical efficacy, and long-term safety data are still lacking. Another major limitation is uncertainty in active-entity attribution: some studies use parent ergosterol, some use ergosterol peroxide or its derivatives, some involve metabolites generated after oral ergosterol exposure, and others use mixed extracts or multi-component systems.

Across these disease areas, mechanistic evidence ranges from biochemical or enzyme-level findings to receptor/pathway-level assays and downstream protein-expression readouts. Relatively stronger mechanistic evidence is available for cholesterol mixed-micelle competition, DHCR7-mediated conversion to brassicasterol, XO/COX-2 enzyme inhibition, LXR-related sterol-homeostasis activity, FOXA1/CPT1A-associated fatty acid oxidation, PPARγ-dependent antifibrotic signaling, and microbiota-mediated AhR activation. By contrast, many PI3K/Akt, NF-κB, MAPK, STAT3, Wnt/β-catenin, TGF-β1/Smad2, Nrf2/HO-1, apoptosis, autophagy, and MDR-related observations are best viewed as model-specific pathway or protein readouts unless supported by direct binding, target engagement, genetic rescue, or structure-based evidence.

Future studies should move from activity discovery toward validation of translationally relevant active entities. This requires parallel comparison of parent ergosterol, ergosterol peroxide, brassicasterol, and major metabolites within the same experimental system, together with parent/metabolite quantification, tissue exposure assessment, dose–exposure–response analysis, and mechanism-directed intervention experiments. Only when the administered compound, *in vivo* active entity, biological effect, and causal mechanism are integrated can the development value of ergosterol-based compounds in specific disease areas be assessed more reliably. To clarify the cellular and tissue contexts underlying these diverse preclinical findings, the principal sites implicated in ergosterol-related studies are summarized in Table 4.

## 7. Formulation and Delivery Strategies

The application of delivery systems in ergosterol-related research mainly serves two purposes. First, delivery platforms enhance ergosterol exposure by improving solubilization, protecting the molecule from degradation during gastrointestinal transit, and ultimately increasing oral absorption and systemic bioavailability. Accordingly, a range of carriers—including liposomes, nanoparticles, microemulsions, micelles, and ferritin cages—has been developed to increase the effective concentration of ergosterol at target sites and enhance its pharmacological effects. Second, ergosterol can serve as a structural sterol, partially replacing cholesterol as a membrane stabilizer. In this role, ergosterol may modulate membrane packing, membrane order, permeability, and release kinetics in a formulation-dependent manner. Because ergosterol itself is bioactive, incorporating it into delivery systems may also provide additive or synergistic effects in certain anticancer and antimicrobial formulations. Nevertheless, ergosterol is chemically less stable than cholesterol and can undergo oxidation or photodegradation; therefore, the long-term stability of ergosterol-containing formulations and their performance under realistic storage and *in vivo* conditions require further systematic evaluation.

### 7.1. Delivery Systems for Improving Ergosterol Exposure and Bioactivity

Although ergosterol exhibits diverse physiological activities, its poor aqueous solubility and instability, including photosensitivity and oxidative instability, result in limited oral bioavailability, thereby limiting systemic exposure and weakening pharmacological efficacy [68,69,103,104]. These constraints are not merely formulation issues; they directly restrict systemic exposure after oral dosing and shape the translational ceiling of ergosterol. To address these bottlenecks, multiple delivery strategies have been explored to improve solubilization, protect ergosterol during gastrointestinal transit, and enhance absorption. Reported ergosterol delivery systems differ in carrier type, payload composition, evaluation endpoint, and extent of *in vivo* validation. Table 5 summarizes the key formulation characteristics, exposure or functional improvement, and major translational limitations of representative delivery platforms. Because dose, formulation composition, analytical method, and pharmacokinetic endpoint differ across studies, the reported bioavailability or exposure improvements should be interpreted as within-study improvements relative to their own controls rather than as direct evidence of superiority between platforms. Representative advances are discussed below.

#### 7.1.1. Liposomes

**Figure 5:**
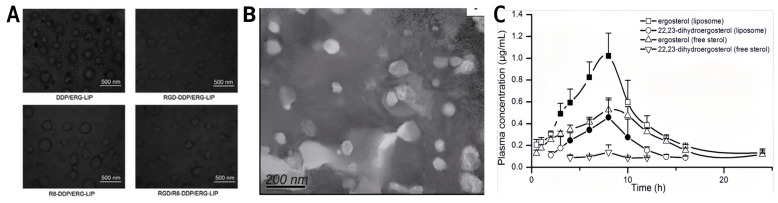
Characterization of FVSL morphology and its enhanced *in vivo* pharmacokinetic profile. (**A**) shows transmission electron microscopy images of four types of liposomes (DDP/ERG-LIP, RGD-DDP/ERG-LIP, R8-DDP/ERG-LIP, RGD/R8-DDP/ERG-LIP) [105]; (**B**) presents transmission electron microscopy images of the FVSL liposome [70]; (**C**) shows the average (+SD) plasma concentration–time curve of ergosterol and 22,23-dihydroergosterol in healthy SD rats (n = 5) after oral administration of a single dose of 20 mL/kg of 5 mg/mL FVSL/free FVS [70]. In panel (**C**), □ represents ergosterol from FVSL, ○ represents 22,23-dihydroergosterol from FVSL, △ represents ergosterol from free FVS, and ▽ represents 22,23-dihydroergosterol from free FVS. Solid symbols, including ■ and ●, indicate time points with significant differences compared with the corresponding free sterol group (*p* < 0.05).

Liposomes are among the most commonly used systems to improve ergosterol delivery efficiency. They have a hydrophilic core and a hydrophobic bilayer, making them suitable for encapsulating hydrophobic molecules such as ergosterol. In a representative targeted design, ergosterol was co-loaded with cisplatin, and the liposomes were further modified with cyclic RGD and octa-arginine (R8) peptides. The preparation typically involves the thin-film dispersion method, combined with post-insertion anchoring of functional peptides through PEG spacers onto the lipid bilayer. Figure 5A shows spherical bilayer structures with a particle size of ~155.2 nm and a surface potential of +4.74 mV, which are associated with improved serum stability and enhanced antitumor performance against A549 lung cancer cells [105]. However, this strategy is not universally effective. On the one hand, the preparation process is relatively complex and may challenge scalability and batch-to-batch reproducibility; on the other hand, the targeting benefit depends heavily on RGD–αvβ3 binding, meaning that tumors with low αvβ3 expression may show limited improvement in targeting and efficacy [105]. The peptide-modified liposomal system was primarily developed as a tumor-targeting delivery platform, with a focus on enhancing cellular uptake and antitumor efficacy rather than on improving oral pharmacokinetics [105].

Traditional liposomes have also been used to encapsulate enoki mushroom sterols (rich in ergosterol). Figure 5B shows the microstructure of FVSL, with spherical liposomes, small particle size, and relatively uniform distribution. As shown in Figure 5C, after oral administration of 100 mg/kg (calculated as total FVS) in SD rats, the FVS liposome formulation (FVSL) increased the relative bioavailability of ergosterol and 22,23-dihydroergosterol to 162.9% and 244.2%, respectively, compared with the corresponding free sterol formulation [70]. Increased exposure was accompanied by improved distribution in key tissues, such as the liver and kidney, reduced IC_50_ values in HepG-2 and A549 cells, and enhanced *in vivo* inhibition [70]. Nevertheless, clear limitations remain: FVSL primarily distributes to the liver and spleen, showing limited effects on some non-hepatic/non-splenic tumors, and it is rapidly cleared *in vivo* within ~4 h, suggesting that frequent administration may be required to maintain effective exposure [70].

Beyond serving as a carrier, ergosterol can also act as a membrane stabilizer for liposomes, partially replacing cholesterol or bile salts. Ergosterol effectively regulates the stability, structure, and membrane properties of soy and egg yolk lecithin liposomes, with membrane-stabilizing performance comparable to, or even better than, cholesterol in certain contexts [106]. In a simulated gastrointestinal environment, ergosterol-stabilized liposomes protected insulin, significantly enhancing its oral absorption and glucose-lowering effects [107]. Additionally, folate-modified ergosterol-based liposomes were used to co-deliver doxorubicin and doxycycline, employing a “chemotherapy–antibiotic–ergosterol” synergistic strategy that suppressed both primary and lung metastatic tumors in triple-negative breast cancer while regulating the tumor microbiome [108]. Ergosterol has also been applied in basic membrane research: ergosterol combined with nystatin forms a composite channel that can act as a “fusion trigger,” enabling controlled delivery of membrane proteins (e.g., β-amyloid peptides) or voltage-gated sodium channels to artificial planar lipid bilayers, providing a useful tool for membrane protein functional studies [109,110]. Overall, liposome-based systems can enhance the bioavailability of ergosterol as a payload, and ergosterol can also serve as a functional membrane component, further optimizing carrier behavior and therapeutic performance.

#### 7.1.2. Nanoparticles

**Figure 6:**
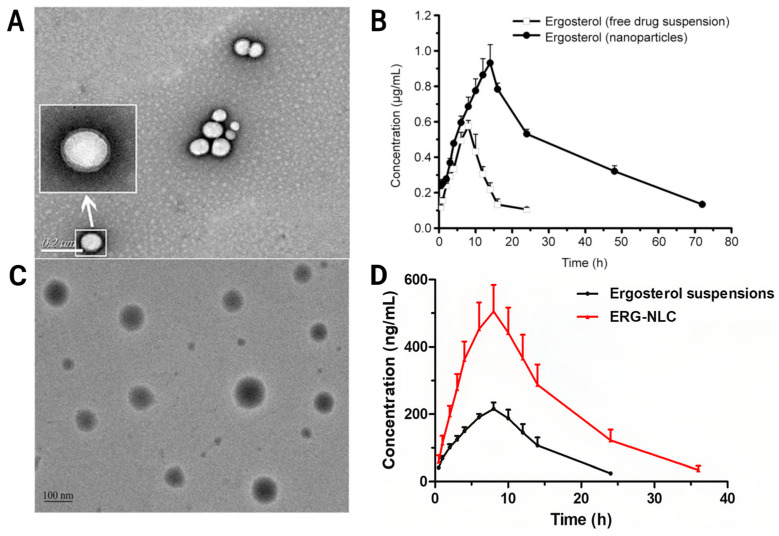
Structural and Functional Validation of Ergosterol Nano-Delivery Systems. (**A**) shows transmission electron microscopy images of NP/Erg [68]. (**B**) presents the average plasma concentration–time curve of ergosterol after oral administration of 50 mg/kg doses of NPs/Erg and free ergosterol suspension to rats [68]. (**C**) shows transmission electron microscopy images of nanoparticle ERG-NLCs [69]. (**D**) presents the plasma concentration–time curve of free ergosterol and nanoparticle ERG-NLCs [69].

Nanoparticles, especially biodegradable polymer nanoparticles and nanostructured lipid carriers (NLCs), have shown potential to enhance the oral bioavailability and therapeutic efficacy of ergosterol, owing to their high loading capacity and tunable release. As shown in Figure 6A, ergosterol-loaded PLGA nanoparticles (NPs/Erg) are spherical or quasi-spherical with relatively uniform distribution and minimal aggregation. As shown in Figure 6B, after oral administration of 50 mg/kg in rats, the PLGA nanoparticle formulation (NPs/Erg) produced a prolonged plasma circulation profile and increased the oral bioavailability of ergosterol by approximately 4.9-fold compared with free ergosterol suspension under the reported conditions [68]. *In vitro*, these nanoparticles exhibited stronger cytotoxicity against U251, MCF-7, and HepG-2 cells than free ergosterol, with IC_50_ reductions of 8.64%, 19.94%, and 26.79%, respectively. *In vivo*, they also increased brain distribution and improved systemic exposure after oral dosing [68]. The improved cytotoxicity may be attributed to endocytosis-mediated uptake and intracellular release that partially bypasses efflux mechanisms. However, long-term storage may lead to aggregation or drug leakage, which represents practical barriers that should be addressed for translation [68].

ERG-NLCs were developed to mitigate leakage from first-generation solid lipid nanoparticles caused by complete lipid crystallization. As shown in Figure 6C, ERG-NLCs exhibit smooth, near-spherical morphology. As shown in Figure 6D, after oral administration of 25 mg/kg in SD rats, ERG-NLCs markedly increased both Cmax and AUC0–∞ relative to raw ergosterol, with a reported relative oral bioavailability of 277.56% [69]. *In vitro* pharmacodynamic tests also showed stronger inhibition of mesangial cell proliferation and extracellular matrix accumulation under high-glucose conditions, highlighting their potential relevance to diabetic nephropathy [69]. Nevertheless, ERG-NLCs may require low-temperature storage, and the preparation process involves high temperatures, which can challenge thermosensitive co-loads; drug-loading capacity is also limited [69].

Nanoparticle systems have also been explored to enhance antibacterial performance. Ergosterol-loaded Prussian blue nanoparticles wrapped in red blood cell membranes form a biomimetic system in which near-infrared activation enables synergistic photothermal–chemical effects. The red blood cell membrane coating extends circulation time and improves targeting to infection sites, thereby enhancing activity against methicillin-resistant *Staphylococcus aureus* and accelerating wound healing [111]. However, this platform introduces practical constraints: fabrication is complex and maximal efficacy depends on external laser irradiation, which may limit broad applicability [111]. Overall, nanoparticle-based strategies can substantially increase ergosterol exposure and efficacy signals, but stability, storage behavior, and operational feasibility should be discussed alongside pharmacodynamic outcomes to strengthen translational credibility [68,69,111].

#### 7.1.3. Microemulsions, Micelles, and Ferritin Cages

**Figure 7:**
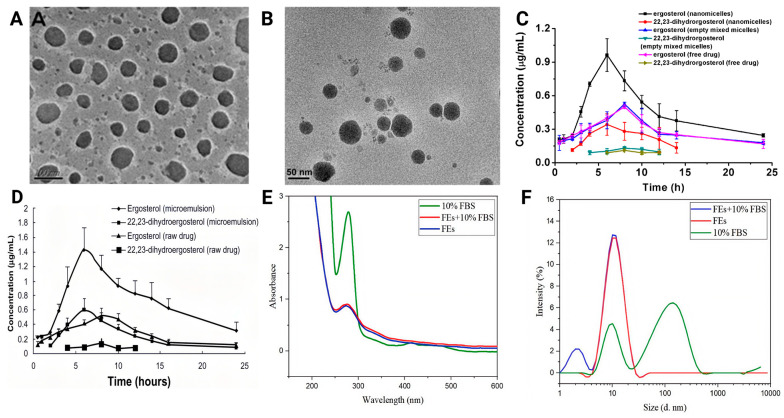
Structural and functional validation of microemulsions, mixed micelles, and ferritin cages. (**A**) shows transmission electron microscopy images of FVSNs mixed micelles [71]. (**B**) presents transmission electron microscopy images of FVSM microemulsions [72]. (**C**) shows the average plasma concentration–time curve of FVS after oral administration of FVSNs and free FVS suspensions at doses of 100 mg/kg [71]. (**D**) presents the plasma concentration–time curve of FVS (ergosterol and 22,23-dihydroergosterol) after oral administration of 100 mg/kg doses of free FVS suspension and FVS-loaded microemulsion (FVSM) [72]. (**E**) shows the UV–visible spectrum of FEs in serum. (**F**) presents the size distribution of FEs in serum [103].

Microemulsions and mixed micelle nanocarriers can effectively increase the solubility and *in vivo* absorption efficiency of ergosterol. For sterols extracted from enoki mushrooms (mainly ergosterol), two representative systems are PVP–phospholipid–sodium cholate mixed micelles (FVSNs) and oil-in-water microemulsions (FVSMs). Figure 7A shows spherical FVSNs micelles with smooth surfaces, while Figure 7B shows FVSM droplets with regular spherical morphology. As shown in Figure 7C, after oral administration of 100 mg/kg (as total FVS) in SD rats, the mixed micellar nanoformulation (FVSNs) produced higher plasma concentrations than free FVS throughout the sampling period; compared with free FVS, the AUC of ergosterol and 22,23-dihydroergosterol increased by approximately 1.55-fold and 2.77-fold, respectively, while Cmax increased by 1.75-fold and 3.43-fold [71]. The advantage of this system is its relatively simple preparation, whereas its sustained-release capacity appears limited, and stability remains insufficiently characterized [71].

Oil-in-water microemulsions (FVSMs) can also markedly enhance oral bioavailability. As shown in Figure 7D, in rats, oral administration of the *Flammulina velutipes* sterol microemulsion (FVSM) markedly improved systemic exposure relative to free FVS, with AUC-based relative oral bioavailability increasing by 2.56-fold for ergosterol and 4.50-fold for 22,23-dihydroergosterol under the reported conditions [72]. Nevertheless, key risks should be stated explicitly: demulsification occurs after ~30 days, stability decreases at higher temperatures, and surfactants such as Cremophor EL and ethanol may irritate the gastrointestinal tract; moreover, the mechanisms underlying *in vivo* distribution and metabolism remain insufficiently clarified [72].

Ferritin cages are protein nanocages that can encapsulate hydrophobic molecules through self-assembly. Encapsulation within recombinant human H-ferritin (rHuHF) produced nanocomposites (FEs) with improved photostability and serum stability of ergosterol, as evidenced by minimal changes in characteristic signals and stable particle size/distribution after serum exposure (Figure 7E,F) [103]. The system exhibited sustained-release behavior during simulated gastrointestinal digestion (≈30% release in gastric fluid and ≈35% in intestinal fluid), whereas the free drug was released more rapidly. In addition, the digesta reduced cholesterol solubility in micelles, suggesting potential cholesterol-lowering effects [103]. However, drug loading is relatively low, which may position ferritin cages more appropriately for functional foods than for therapeutic dosing [103]. The observed reduction in micellar cholesterol solubility suggests a potential cholesterol-lowering implication, but this inference should be interpreted cautiously because the current evidence remains formulation- and model-dependent [76,99].

In summary, microemulsion/micelle systems and ferritin cages can enhance ergosterol bioavailability, thereby improving physiological activity. Yet, their applicability depends on excipient safety, storage stability, and clarity of *in vivo* fate rather than pharmacokinetic gains alone [71,72,77,103]. To overcome ergosterol’s fundamental drawbacks—poor water solubility, low stability, and extremely low oral bioavailability—delivery systems such as liposomes, nanoparticles, microemulsions, micelles, and ferritin cages have been widely studied [68,69,103,104]. Moving forward, long-term stability, *in vivo* metabolic fate, and potential immunogenicity of these nanocarriers should be systematically evaluated, and the preparation processes should be advanced toward greener, standardized, and scalable manufacturing to support meaningful clinical translation [68,69,103,104].

### 7.2. Ergosterol as a Functional Membrane Component in Delivery Systems

Ergosterol can be incorporated into selected membrane-based delivery systems as a cholesterol-like sterol or functional membrane component. In this context, its role differs from that of ergosterol as a poorly soluble payload: it is mainly used to modulate carrier membrane organization, vesicle stability, encapsulation behavior, and drug-release profiles. Molecular dynamics studies of sterol-containing niosome bilayers indicate that sterol structure can influence Span60 bilayer organization, area per molecule, thickness, compressibility, and hydrogen-bonding patterns; therefore, the effect of ergosterol should be interpreted as formulation-dependent rather than as universally superior to cholesterol [112]. Table 6 summarizes the reported effects of ergosterol incorporation on delivery-system properties and drug performance.

#### 7.2.1. Ergosterol as a Membrane Stabilizer

The structural similarity between ergosterol and cholesterol allows ergosterol to replace cholesterol or act as an alternative helper lipid in some vesicular systems. However, the stabilizing effect of ergosterol depends on the surfactant type, sterol ratio, co-lipids, payload properties, and release environment. Thus, ergosterol-containing systems are more appropriately described as membrane-modulating carriers rather than universally stronger membrane-stabilized systems [112].

Several ergosterol-containing niosomal systems have been reported for anticancer and gene-delivery applications. In methotrexate-loaded pH-responsive niosomes, the formulation containing ergosterol showed a particle size of 176.7 ± 3.4 nm, zeta potential of −31.5 ± 2.6 mV, encapsulation efficiency of 76.9 ± 2.5%, pH-responsive behavior at pH 5.4 and 7.4, and enhanced cytotoxicity against MCF-7 cells compared with free methotrexate [113]. Similarly, carfilzomib-loaded ergosterol/CHEMS pH-responsive niosomes achieved an encapsulation efficiency of 89.82%, with particle size increasing from 323 nm in empty niosomes to 334 nm after drug loading. The formulation released 74.39% of carfilzomib at pH 5.4 and 54.55% at pH 7.4, supporting acidic pH-responsive release [114]. Ergosterol-based niosomes have also been used to encapsulate thymoquinone or *Carum carvil* extract, providing high encapsulation efficiency, controlled release, enhanced cytotoxicity, G2/M-phase arrest, and migration inhibition in MCF-7 breast cancer cells [115]. Beyond small-molecule delivery, magnetic niosomes containing ergosterol have been applied to plasmid DNA delivery, showing nanoscale size, positive zeta potential, 72% plasmid loading, and increased gene expression under an external magnetic field [116].

#### 7.2.2. Ergosterol-Containing Systems for Improving Drug Performance

When ergosterol is incorporated into delivery systems, the improved biological effect may result from multiple factors, including altered membrane organization, improved payload encapsulation, controlled release, enhanced cellular uptake, and the intrinsic biological activity of ergosterol itself. Therefore, additive or synergistic effects should be interpreted within each formulation design and ideally supported by comparisons with blank carriers, cholesterol-based carriers, free drugs, single-agent controls, and dose-matched combinations.

In anticancer delivery, ergosterol has been explored not only as a membrane component but also as a self-assembling bioactive carrier. Self-assembled ergosterol nanoparticles loaded with chlorin e6 improved water solubility and stability, promoted intracellular ROS generation, enhanced phototoxicity against 4T1 and MCF-7 cancer cells, and produced stronger *in vivo* antitumor efficacy than either ergosterol nanoparticles or chlorin e6-mediated photodynamic therapy alone [117]. In extract-based systems, niosomes loaded with *Ganoderma lucidum* and *Lentinus edodes* extracts showed enhanced anticancer and antimicrobial effects; however, this system used cholesterol-containing niosomes, while ergosterol was identified as one of the bioactive constituents in the mushroom extracts rather than as the carrier sterol [118].

Ergosterol-containing systems have also been investigated for local antifungal delivery. In curcumin-loaded mucoadhesive liquid-crystalline systems, oleic acid/ergosterol was used as the oil phase, PPG-5-CETETH-20 as the surfactant, and chitosan dispersion as the aqueous phase. This formulation supported controlled release, mucoadhesion, membrane interaction, and improved anti-*Candida* performance in vulvovaginal candidiasis models [119]. Lipo-niosome systems co-loading amphotericin B and thymus essential oil also improved antifungal performance and reduced host-cell toxicity compared with free-drug combinations [120]. However, these amphotericin B-based systems should be distinguished from ergosterol-containing carriers because their antifungal activity mainly involves drug interaction with fungal membrane ergosterol rather than ergosterol functioning as a formulation component.

For bioactive molecule delivery, phytosterol-based liposomes and niosomes have been evaluated to improve peptide stability and bioaccessibility. In goat milk whey protein peptide delivery, several phytosterols, including ergosterol, β-sitosterol, mixed phytosterols, and stigmasterol, were compared as cholesterol substitutes. The best-performing niosomes in that study were prepared with β-sitosterol rather than ergosterol; therefore, this evidence supports sterol-dependent carrier design but should not be interpreted as direct evidence for ergosterol-specific superiority [121].

Overall, ergosterol-containing membrane systems provide a useful strategy for tuning carrier properties and improving selected payload performance. Their translational value depends on formulation-specific evidence, including particle size, PDI, zeta potential, encapsulation efficiency, drug loading, release kinetics, storage stability, biological efficacy, toxicity, and scalability. Future studies should use matched cholesterol-containing controls and should distinguish the effects of carrier structure, payload activity, and the intrinsic bioactivity of ergosterol.

### 7.3. Comparative Limitations and Translational Considerations

Current ergosterol delivery systems have shown potential to improve dispersion, stability, release behavior, and systemic exposure. However, direct comparisons across platforms remain limited because liposomes, PLGA nanoparticles, nanostructured lipid carriers, mixed micelles, microemulsions, and ferritin cages differ in payload composition, administered dose, animal model, analytical method, pharmacokinetic sampling window, and pharmacokinetic endpoint. Thus, reported bioavailability gains are most appropriately viewed as within-study improvements relative to matched controls rather than as direct cross-platform rankings. For example, the approximately 4.9-fold bioavailability increase reported for PLGA nanoparticles and the 277.56% relative oral bioavailability reported for ERG-NLCs were obtained under different formulation designs, doses, pharmacokinetic protocols, and endpoint definitions; these values should not be used to rank platform superiority directly. In addition, several studies using FVSL, FVSNs, and FVSMs were based on mixed sterol fractions from *Flammulina velutipes*, in which ergosterol and 22,23-dihydroergosterol are simultaneously present; these findings should not be considered fully equivalent to purified ergosterol formulations.

Based on the available evidence, PLGA nanoparticles and nanostructured lipid carriers appear to be the most promising platforms for improving systemic exposure of ergosterol as an active compound, although their translational value remains formulation-dependent. PLGA nanoparticles provide prolonged circulation, enhanced oral exposure, and improved tissue distribution, including brain exposure, but their development may be constrained by organic solvent-based preparation, aggregation or drug leakage during storage, and the lack of metabolite-resolved pharmacokinetic data. ERG-NLCs show high encapsulation efficiency and a clear oral exposure advantage, making them attractive for oral delivery; however, their relatively low drug-loading capacity, heat-based preparation process, potential storage-temperature sensitivity, and absence of tissue-distribution data remain important limitations. Liposomes, mixed micelles, and microemulsions also improve oral exposure, but their current evidence is often based on mixed sterol payloads, and their further development may be limited by rapid clearance, incomplete long-term stability data, high surfactant or co-solvent content, gastrointestinal irritation, dilution instability, or demulsification during storage. Ferritin cages improve light stability, serum stability, and simulated gastrointestinal release, but their low loading capacity and lack of *in vivo* pharmacokinetic validation suggest greater suitability for functional food applications than for therapeutic dosing.

Scale-up feasibility should be evaluated together with pharmacokinetic performance. For liposomes and peptide-modified liposomes, manufacturing complexity, sterilization, ligand-conjugation efficiency, and batch-to-batch reproducibility are major barriers. For PLGA nanoparticles, residual organic solvent, particle aggregation, drug leakage, and reproducible control of particle size, PDI, encapsulation efficiency, and drug loading require systematic optimization. For NLCs, lipid polymorphism, crystallization behavior, thermal processing, storage temperature, and long-term leakage should be assessed under accelerated and real-time stability conditions. For microemulsions, mixed micelles, and ferritin cages, excipient safety, dilution stability, protein-source consistency, immunogenicity, production cost, and loading capacity require further evaluation. Therefore, the most promising ergosterol delivery system should not be defined solely by apparent bioavailability enhancement, but by the combined evidence for loading capacity, release behavior, storage stability, excipient safety, scalability, metabolite-resolved pharmacokinetics, tissue distribution, and disease-relevant pharmacodynamic validation. For ergosterol-containing membrane systems, the role of ergosterol as a delivered active compound, functional membrane component, or pharmacological contributor should also be distinguished, and claims of additive or synergistic effects should be supported by matched blank-carrier, cholesterol-containing carrier, single-agent, and equivalent-dose combination controls.

**Table 6:** Impact of Ergosterol as a Functional Membrane Component on Delivery Systems and Drug Performance.

Aspect	Performance & Role	Key Mechanism/Evidence	References
Effect on the delivery system	Modulates niosome membrane organization	Sterol structure influences Span60 bilayer organization, molecular packing, membrane thickness, compressibility, and hydrogen-bonding patterns; the effect of ergosterol is formulation-dependent.	[112]
	Supports pH-responsive anticancer nanocarriers	MTX-loaded ergosterol-modified niosomes showed nanoscale size, negative zeta potential, 76.9 ± 2.5% encapsulation efficiency, and pH-responsive release behavior.	[113]
	Enables controlled acidic release	CFZ-loaded ergosterol/CHEMS niosomes showed 89.82% encapsulation efficiency, particle size of 334 nm after loading, and higher CFZ release at pH 5.4 than pH 7.4.	[114]
	Supports macromolecule encapsulation and magnetic-field-assisted delivery	Magnetic ergosterol-containing niosomes achieved nanoscale size, positive zeta potential, 72% plasmid loading, and increased gene expression under an external magnetic field.	[116]
Effect on drugs	Enhances anticancer performance of phytochemical-loaded niosomes	Ergosterol-based niosomes loaded with TQ or *Carum carvil* extract showed high encapsulation efficiency, controlled release, enhanced cytotoxicity, G2/M arrest, and migration inhibition in MCF-7 cells.	[115]
	Functions as a self-assembled bioactive carrier	Self-assembled ergosterol nanoparticles loaded with chlorin e6 improved solubility, stability, ROS generation, phototoxicity, and *in vivo* antitumor efficacy.	[117]
	Improves local antifungal delivery performance	In CUR-loaded mucoadhesive liquid-crystalline systems, oleic acid/ergosterol was used as the oil phase to support controlled release, mucoadhesion, membrane interaction, and anti-*Candida* performance.	[119]

## 8. Translational Gaps and Future Perspectives

Although ergosterol and its related compounds have shown broad bioactivities across disease models and delivery systems, several key barriers still limit their translational development. First, active-entity attribution remains insufficiently resolved. Some studies investigate parent ergosterol, some focus on ergosterol peroxide, whereas others involve brassicasterol [11], ERG1, ERG2 [12], hydroxylated metabolites [67], or mixed extracts. Because orally administered ergosterol can undergo absorption, metabolic conversion, and elimination, *in vivo* effects cannot always be attributed directly to unchanged parent ergosterol. Future studies should therefore distinguish parent ergosterol, ergosterol peroxide, and major metabolites within the same experimental system, while integrating parent/metabolite-resolved quantification and mechanistic validation to clarify the active entity responsible for the observed effects.

Second, the current pharmacokinetic evidence remains insufficient to support definitive exposure–response interpretation. Available studies indicate that free ergosterol has limited oral absorption, substantial fecal elimination, and metabolic conversion; stable isotope-labeled studies also suggest that brassicasterol exposure may exceed parent ergosterol exposure after oral administration [12,63,64]. However, complete mass-balance studies, tissue distribution studies, and systematic dose–exposure–response evaluations remain limited. Future studies should use validated LC–MS/MS methods and stable isotope-tracing strategies to quantify parent compounds and major metabolites in plasma, tissues, feces, urine, and bile, thereby determining whether the reported disease-related effects can be achieved at pharmacologically plausible exposure levels.

Third, disease-oriented evidence remains largely preclinical. Anticancer [1] and metabolic regulation studies [3] are relatively abundant and mechanistically diverse, whereas inflammatory organ injury [2], neuroprotection [6], gut microbiota modulation [101], osteoarthritis [102], and other emerging activities remain at earlier exploratory stages. Most studies rely on cellular experiments or animal models, and human pharmacokinetic, clinical efficacy, and long-term safety data are lacking. In addition, differences in dose, administration route, compound form, and disease endpoints limit cross-study comparison. Future disease-oriented studies should move from activity discovery toward translational validation by establishing dose–exposure–response relationships, target-tissue exposure, causal mechanism, and safety margins.

Fourth, formulation and delivery studies have demonstrated potential to improve ergosterol dispersion, stability, oral exposure, and delivery performance, but unified evaluation across platforms is still limited [68,69,103,104]. Current delivery systems have two distinct objectives. One strategy formulates ergosterol as the active compound to improve solubilization, absorption, and *in vivo* exposure. The other uses ergosterol as a membrane stabilizer or functional carrier component to improve the delivery performance of other drugs or composite carriers. The former should be evaluated using solubility, bioaccessibility, Cmax, Tmax, AUC, tissue distribution, and parent/metabolite exposure, whereas the latter should be evaluated using membrane stability, leakage rate, release kinetics, direct comparison with cholesterol-containing systems, co-loaded drug efficacy, and synergy validation. Future formulation studies should avoid relying only on “enhanced bioavailability” or “improved delivery efficiency” as final conclusions, and should instead systematically link formulation parameters, pharmacokinetic exposure, and pharmacodynamic endpoints.

Finally, the translational development pathway of ergosterol-based compounds remains to be clarified. Parent ergosterol may be more suitable as a functional ingredient, metabolic modulator, cholesterol-homeostasis regulator, or formulation excipient. Ergosterol peroxide may be more relevant as an anticancer lead compound or radiosensitization-related candidate. Brassicasterol and other metabolites may help explain *in vivo* effects after oral ergosterol exposure. These different development directions require different evaluation standards, including pharmacokinetic exposure, target-tissue distribution, toxicity window, long-term safety, formulation stability, and manufacturing scalability. Only when active entity, pharmacokinetic exposure, disease-model efficacy, and formulation strategy are clearly connected can the translational value of ergosterol-based compounds be reliably assessed.

## 9. Conclusions

Ergosterol is a biologically and pharmacologically important fungal sterol that serves not only as a precursor of vitamin D_2_ but also as a molecular link among fungal membrane biology, metabolic regulation, inflammatory control, disease intervention, and delivery-system design. Current evidence indicates that ergosterol, ergosterol peroxide, and related metabolites exhibit diverse preclinical activities in cancer, metabolic disorders, inflammatory organ injury, neuroprotection, and gut microbiota-related disease models. These activities arise from chemically distinct entities, including parent ergosterol, ergosterol peroxide, and metabolism-related sterols, and their interpretation depends on compound identity, administration route, systemic exposure, and disease model.

Overall, the translational potential of ergosterol-based compounds depends on three key issues: whether the active entity is clearly defined, whether *in vivo* exposure is sufficient to support the observed pharmacological effects, and whether formulation strategies can enhance effective exposure in a stable, controllable, and safe manner. Future studies should integrate chemical and biological characterization, pharmacokinetics, disease mechanisms, and delivery-system evaluation to determine whether ergosterol-based compounds are more suitable as functional ingredients, drug leads, formulation excipients, or scaffolds for derivative development.

## Figures and Tables

**Table 1:** Summary of available *in vivo* pharmacokinetic and exposure data for free and formulated ergosterol.

Study/Formulation	Model	Dose/Route	Measured Entity	Main PK or Exposure Finding	Exposure Change	Key Limitation	Ref.
Radiolabeled free ergosterol	Rats	Oral ergosterol-C14 in corn oil; IV ergosterol-C14 in selected experiments	Total ergosterol-derived radioactivity	Only a small fraction of oral radioactivity was absorbed; most remained in the gastrointestinal tract and feces. Absorbed radioactivity was mainly detected in liver, lung, spleen, and adrenal glands	Approximately 3–5% oral absorption in normal rats; 2.2–4.8% lymphatic absorption in thoracic duct fistula rats	Parent compound and metabolites were not resolved; no conventional Cmax, Tmax, AUC, or t1/2 values	[63]
Free ergosterol	Male SD rats	100 mg/kg, oral gavage	Parent ergosterol; ERG1 and ERG2 identified qualitatively	Parent ergosterol was detected in plasma, urine, and feces; fecal excretion was the dominant elimination route	Cmax 2.27 ± 0.19 µg/mL; Tmax 8.00 ± 1.18 h; AUC0–36 h 22.29 ± 5.08 µg·h/mL; fecal recovery 62.5%; urinary recovery 3.2%	Limited tissue distribution and incomplete mass-balance analysis	[12]
Stable isotope-labeled ergosterol	SHRSP rats	100 mg/kg ergosterol-d1, oral administration	Ergosterol-d1 and brassicasterol-d1	Ergosterol-d1 and brassicasterol-d1 were simultaneously detected in serum after oral dosing	Ergosterol-d1 Cmax 0.552 ± 0.138 µg/mL; AUC0–36 h 3.88 ± 1.06 µg·h/mL; brassicasterol-d1 peak serum concentration was approximately 3-fold higher than ergosterol-d1	Serum-focused study; no tissue distribution or mass-balance data	[64]
PLGA nanoparticles	Rats; tissue distribution in mice	50 mg/kg, oral administration	Parent ergosterol	PLGA nanoparticles prolonged systemic exposure and improved tissue distribution, especially brain exposure	AUC0–72 h increased from 6.35 ± 0.95 to 31.12 ± 4.76 µg·h/mL; oral bioavailability increased approximately 4.90-fold	No metabolite-resolved PK; formulation stability concerns remain	[68]
Nanostructured lipid carriers	SD rats	25 mg/kg, oral administration	Parent ergosterol	ERG-NLCs increased Cmax and AUC compared with raw ergosterol suspension	AUC0–∞ increased from 2991.90 ± 385.28 to 8304.29 ± 1277.73 ng·h/mL; relative bioavailability 277.56%	No tissue distribution or metabolite-resolved analysis	[69]
*Flammulina velutipes* sterol liposomes	SD rats; tissue distribution in mice	100 mg/kg total FVS, oral administration	Ergosterol and 22,23-dihydroergosterol	Liposomal encapsulation increased systemic exposure and promoted early liver and spleen distribution	Relative bioavailability increased to 162.9% for ergosterol and 244.2% for 22,23-dihydroergosterol	Mixed sterol formulation; short tissue-distribution window	[70]
Mixed micelles	SD rats; tissue distribution in mice	Single oral dose of FVS	Ergosterol and 22,23-dihydroergosterol	Mixed micelles increased plasma exposure and altered tissue distribution compared with free FVS	Relative bioavailability approximately 154.54% for ergosterol and 276.22% for 22,23-dihydroergosterol	Mixed sterol formulation; dose reporting requires careful interpretation	[71]
Oil-in-water microemulsion	Rats	100 mg/kg FVS, oral administration	Ergosterol and 22,23-dihydroergosterol	Microemulsion markedly improved systemic exposure compared with free FVS	Relative bioavailability increased 2.56-fold for ergosterol and 4.50-fold for 22,23-dihydroergosterol	No tissue distribution; stability depends on formulation conditions	[72]

**Table 2:** Anticancer effects of ergosterol, ergosterol peroxide, and related derivatives.

Cancer Type	Compound	Main Mechanism/Effect	Associated Pathway/Protein Readouts	References
Breast Cancer	Ergosterol	Upregulates FOXO3; inhibits cancer cell proliferation	FOXO3-related apoptotic mediators; AKT/GSK-3β/β-catenin-related readouts	[1,13]
	Ergosterol peroxide and derivatives	Inhibits proliferation and metastasis	PI3K/AKT/mTOR-, NF-κB-, and STAT3-related readouts	[14,83,87]
	Ergosterol peroxide (TNBC)	Induces mitochondrial dysfunction & ROS accumulation	Mitochondrial function, ROS	[84]
Colorectal Cancer	Ergosterol and its metabolites	LXR-related activity; altered cholesterol-homeostasis targets; inhibited proliferation/metastasis-related phenotypes	LXR-related activity; ABCA1/ABCG1 expression	[15,80]
Prostate Cancer	Ergosterol peroxide	Promotes cancer cell apoptosis	DR5/FADD/caspase-8/3 signaling	[82,95]
Ovarian Cancer	Ergosterol peroxide	Inhibits cancer cell proliferation and invasion	β-catenin- and STAT3-related readouts	[88]
Liver Cancer	Ergosterol peroxide	Induces cancer cell death	pAKT, c-Myc, and FOXO3-associated apoptotic signaling	[17]
Lung Cancer	Ergosterol peroxide	Inhibits proliferation and metastasis; induces apoptosis/autophagy; inhibits NLRP3-related inflammatory activity	Mitochondria, ROS, autophagy, NLRP3	[85]
Cervical Cancer	Ergosterol peroxide	Radiosensitizer (low toxicity to normal cells)	Radiation sensitization	[86]
Bladder Cancer	Ergosterol (brassicasterol)	Inhibits cancer development	Cell cycle, inflammation	[92,93]

**Table 3:** Representative anti-inflammatory and protective effects of ergosterol in lung and nervous-system models.

Site of Action	Type of Action	Disease/Model	Associated Mechanism/Readouts	Main Outcome	References
Lung	Protection against lung injury	LPS-induced acute lung injury	Reduces NF-κB-, COX-2-, and iNOS-related inflammatory readouts; decreases TNF-α and IL-6	Alleviates lung edema and pathological damage	[2]
		Cigarette smoke-induced COPD	Reduces JAK3/STAT3/NF-κB-related inflammatory signaling; increases SOD/CAT and decreases MDA	Mitigates disease progression	[97]
Nervous	Anti-neuroinflammation	LPS-induced neuroinflammation; Bisphenol A-induced neurotoxicity	Reduces NF-κB-, AKT-, and MAPK-related inflammatory readouts and pro-inflammatory cytokine production	Alleviates neuroinflammation and injury	[16,99]
	Neuroprotection and repair	Oxidative stress (e.g., TNF-α-induced injury)	Increases Akt-related phosphorylation readouts, enhances antioxidant enzyme activity, and reduces ROS	Reduces neuronal oxidative stress	[6]
		HT-22 cells	Alters EGR1 and Grin2b expression.	Maintains neuronal excitability balance	[6]
		PC12 cells	Ergosterol peroxide promotes NGF-mediated neurite outgrowth	Promotes neuronal repair and regeneration	[89]

**Table 4:** Principal cellular and tissue sites implicated in ergosterol-related preclinical studies.

Representative Entity	Disease/Pathological Context	Main Cellular/Tissue Site Involved	Ref.
Ergosterol	Cancer	Tumor cells, tumor-associated sterol homeostasis, apoptotic machinery, and drug-efflux systems	[1,13,15,79,80,81,92,93]
Ergosterol	Type 2 diabetes and glucose dysregulation	Skeletal muscle glucose-uptake machinery, insulin-responsive tissues, and renal tissue	[3,73]
Ergosterol	Diabetic nephropathy and renal metabolic injury	Renal inflammatory microenvironment, tubular epithelial cells, mesangial cells, and ECM-regulating compartments	[74,75,76]
Ergosterol and ergosterol-enriched extracts	Cholesterol and lipid metabolism	Intestinal mixed micelles, intestinal epithelial transport, hepatic cholesterol metabolism, and fecal sterol excretion	[4,62,77]
Ergosterol-containing interventions	Uric acid regulation and gouty nephropathy	Xanthine oxidase/COX-2-related systems, urate transport machinery, renal tissue, and inflammatory microenvironment	[5,96]
Ergosterol	Acute lung injury and COPD-related lung injury	Pulmonary inflammatory cells, lung parenchyma, oxidative-stress systems, and cytokine-producing compartments	[2,97]
Ergosterol	Hepatic fibrosis and drug-induced renal cell injury	Hepatic stellate cells, renal tubular epithelial-like cells, oxidative-stress systems, and autophagy/apoptosis-related compartments	[7,100]
Ergosterol	Neuroinflammation and neuronal injury	Microglia, hippocampal neurons, oxidative-stress systems, and neuronal excitability-related compartments	[6,16,99]
Ergosterol	Gut–brain axis, intestinal dysfunction, and osteoarthritis	Intestinal barrier, gut microbiota–metabolite axis, hippocampal inflammatory environment, and articular cartilage	[100,101,102]
Ergosterol peroxide and derivatives	Cancer	Tumor-cell mitochondria, ROS-generating systems, apoptotic/autophagic machinery, invasion-related compartments, and radiosensitivity-related pathways	[14,15,17,82,83,84,85,86,87,88,94,95]
Ergosterol peroxide	Skin inflammation	Keratinocytes, oxidative-stress systems, inflammatory cytokine compartments, and skin barrier-related proteins	[90]
Ergosterol peroxide	Renal fibrosis	Renal fibroblasts, ECM-producing compartments, and fibrosis-associated signaling systems	[91]
Ergosterol peroxide and related sterols	Neuronal repair	PC12 neuronal differentiation system and NGF-responsive neurite outgrowth machinery	[89]
Brassicasterol and other ergosterol-related metabolites	Metabolism-related interpretation after oral ergosterol exposure	Systemic circulation, sterol metabolic conversion, and metabolite-associated tissue exposure	[11,12,64,92,93]

**Table 5:** Comparative formulation characteristics and translational limitations of ergosterol delivery systems.

Delivery Platform	Key Formulation Features	Main Improvement	Main Limitation	Ref.
Liposomes	FVSL: ~108 nm; EE 71.3% for ergosterol and 69.0% for 22,23-dihydroergosterol	Oral bioavailability increased to 162.9% and 244.2%, respectively; liver/spleen distribution increased	Mixed sterol payload; rapid elimination; limited long-term stability data	[70]
PLGA nanoparticles	NPs/Erg: 156.9 nm; zeta potential −19.27 mV; EE 76.29%; DL 10.88%	AUC increased ~4.90-fold; prolonged circulation; brain exposure improved	Organic solvent-based preparation; aggregation/leakage risk; no metabolite-resolved PK	[68]
Nanostructured lipid carriers	ERG-NLCs: 81.39 nm; zeta potential −30.77 mV; EE 92.95%; DL 6.51%	Relative oral bioavailability 277.56%; stronger activity in high-glucose mesangial cells	Limited loading capacity; heat-based preparation; no tissue distribution data	[69]
Mixed micelles	FVSNs: ~115.6 nm; EE ~76.6%; DL not reported	Relative bioavailability 154.54% for ergosterol and 276.22% for 22,23-dihydroergosterol	Mixed sterol payload; limited stability data; dose comparability requires caution	[71]
Microemulsions	FVSMs: ~22.9 nm; PDI 0.31; EE 81.1%; FVS solubility 0.680 mg/mL	Relative bioavailability increased 2.56-fold and 4.50-fold for the two major sterols	High surfactant content; no tissue distribution data; stability depends on formulation conditions	[72]
Ferritin cages	FEs: ~17 ergosterol molecules/ferritin; EE 27.28%; DL 1.63%	Improved light stability, serum stability, and simulated gastrointestinal release	Not an *in vivo* PK study; low loading; systemic exposure not established	[103]

## Data Availability

No new data were created or analyzed in this study. Data sharing is not applicable to this article.

## References

[1] Li X., Wu Q., Xie Y., Ding Y., Du W.W., Sdiri M., Yang B.B. (2015). Ergosterol Purified from Medicinal Mushroom *Amauroderma rude* Inhibits Cancer Growth in Vitro and in Vivo by Up-Regulating Multiple Tumor Suppressors. Oncotarget.

[2] Zhang S., Xu L., Li A., Wang S. (2015). Effects of Ergosterol, Isolated from Scleroderma Polyrhizum Pers., on Lipopolysaccharide-Induced Inflammatory Responses in Acute Lung Injury. Inflammation.

[3] Xiong M., Huang Y., Liu Y., Huang M., Song G., Ming Q., Ma X., Yang J., Deng S., Wen Y. (2018). Antidiabetic Activity of Ergosterol from Pleurotus Ostreatus in KK-Ay Mice with Spontaneous Type 2 Diabetes Mellitus. Mol. Nutr. Food Res..

[4] He W.-S., Cui D., Li L., Tong L.-T., Rui J., Li H., Zhang H., Liu X. (2019). Cholesterol-Reducing Effect of Ergosterol Is Modulated via Inhibition of Cholesterol Absorption and Promotion of Cholesterol Excretion. J. Funct. Foods.

[5] Zhou H.B., Feng L.J., Weng X.H., Wang T., Lu H., Bian Y.B., Huang Z.Y., Zhang J.L. (2024). Inhibition Mechanism of Cordycepin and Ergosterol from Cordyceps Militaris Link. against Xanthine Oxidase and Cyclooxygenase-2. Int. J. Biol. Macromol..

[6] Sillapachaiyaporn C., Mongkolpobsin K., Chuchawankul S., Tencomnao T., Baek S.J. (2022). Neuroprotective Effects of Ergosterol against TNF-α-Induced HT-22 Hippocampal Cell Injury. Biomed. Pharmacother..

[7] Qin Z., Xie L., Wang Y., Zhang N., Bi H., Song M., Xu C. (2025). Ergosterol Protects Canine MDCK Cells from Gentamicin-Induced Damage by Modulating Autophagy and Apoptosis. Metabolites.

[8] Liu Z., Deng M., Qu Y., Liang N., Zhao L. (2023). An Efficient Extraction Method for Ergosterol from Lentinus Edodes Stem by Ultrasonic-Assisted Natural Deep Eutectic Solvent. Microchem. J..

[9] Medina M.E., Iuga C., Trigos Á. (2016). Mechanism and Kinetics of the Oxidative Damage to Ergosterol Induced by Peroxyl Radicals in Lipid Media: A Theoretical Quantum Chemistry Study. J. Phys. Org. Chem..

[10] Baur A.C., Kühn J., Brandsch C., Hirche F., Stangl G.I. (2019). Intake of Ergosterol Increases the Vitamin D Concentrations in Serum and Liver of Mice. J. Steroid Biochem. Mol. Biol..

[11] Kuwabara N., Kanda J., Sato S., Nakagawa S. (2025). Impact of Daily High Ergosterol Intake for 14 Weeks in Ovariectomized Rats on Cholesterol and Vitamin D3 Biosynthesis Pathways. Biol. Pharm. Bull..

[12] Zhao Y.-Y., Cheng X.-L., Liu R., Ho C.C., Wei F., Yan S.-H., Lin R.-C., Zhang Y., Sun W.-J. (2011). Pharmacokinetics of Ergosterol in Rats Using Rapid Resolution Liquid Chromatography–Atmospheric Pressure Chemical Ionization Multi-Stage Tandem Mass Spectrometry and Rapid Resolution Liquid Chromatography/Tandem Mass Spectrometry. J. Chromatogr. B.

[13] Nilkhet S., Vongthip W., Lertpatipanpong P., Prasansuklab A., Tencomnao T., Chuchawankul S., Baek S.J. (2024). Ergosterol Inhibits the Proliferation of Breast Cancer Cells by Suppressing AKT/GSK-3beta/Beta-Catenin Pathway. Sci. Rep..

[14] Kang J.-H., Jang J.-E., Mishra S.K., Lee H.-J., Nho C.W., Shin D., Jin M., Kim M.K., Choi C., Oh S.H. (2015). Ergosterol Peroxide from Chaga Mushroom (Inonotus Obliquus) Exhibits Anti-Cancer Activity by down-Regulation of the β-Catenin Pathway in Colorectal Cancer. J. Ethnopharmacol..

[15] Taank Y., Randhawa V., Agnihotri N. (2024). Ergosterol and Its Metabolites as Agonists of Liver X Receptor and Their Anticancer Potential in Colorectal Cancer. J. Steroid Biochem. Mol. Biol..

[16] Sun P., Li W., Guo J., Peng Q., Ye X., Hu S., Liu Y., Liu W., Chen H., Qiao J. (2023). Ergosterol Isolated from Antrodia Camphorata Suppresses LPS-Induced Neuroinflammatory Responses in Microglia Cells and ICR Mice. Molecules.

[17] Li X., Wu Q., Bu M., Hu L., Du W.W., Jiao C., Pan H., Sdiri M., Wu N., Xie Y. (2016). Ergosterol Peroxide Activates Foxo3-Mediated Cell Death Signaling by Inhibiting AKT and c-Myc in Human Hepatocellular Carcinoma Cells. Oncotarget.

[18] Adler J.H., Young M., Nes W.R. (1977). Determination of the Absolute Configuration at C-20 and C-24 of Ergosterol in Ascomycetes and Basidiomycetes by Proton Magnetic Resonance Spectroscopy. Lipids.

[19] McIntosh A.L., Atshaves B.P., Gallegos A.M., Storey S.M., Reibenspies J.H., Kier A.B., Meyer E., Schroeder F. (2008). Structure of Dehydroergosterol Monohydrate and Interaction with Sterol Carrier Protein-2. Lipids.

[20] Sun S., Gao Y., Ling X., Lou H. (2005). The Combination Effects of Phenolic Compounds and Fluconazole on the Formation of Ergosterol in Candida Albicans Determined by High-Performance Liquid Chromatography/Tandem Mass Spectrometry. Anal. Biochem..

[21] Román-Hidalgo C., Villar-Navarro M., Falcón-García G.E., Carbonero-Aguilar M.P., Bautista-Palomas J.D., Bello-López M.A., Martín-Valero M.J., Fernández-Torres R. (2021). Selective, Rapid and Simultaneous Determination of Ergosterol and Ergocalciferol in Mushrooms by UPLC-Q-TOF-MS. J. Pharm. Biomed. Anal..

[22] Heleno S.A., Diz P., Prieto M.A., Barros L., Rodrigues A., Barreiro M.F., Ferreira I.C. (2016). Optimization of Ultrasound-Assisted Extraction to Obtain Mycosterols from *Agaricus bisporus* L. with Response Surface Methodology and Comparison with Conventional Soxhlet Extraction. Food Chem..

[23] Hammann S., Vetter W. (2016). Method Development for the Determination of Free and Esterified Sterols in Button Mushrooms (*Agaricus bisporus*). J. Agric. Food Chem..

[24] Chen Z., Yuan X., Buchanan P., Quek S.Y. (2020). Isolation and Determination of Lipophilic Mycochemicals from a New Zealand Edible Native Mushroom Hericium Novae-Zealandiae. J. Food Compos. Anal..

[25] Krzyczkowski W., Malinowska E., Suchocki P., Kleps J., Olejnik M., Herold F. (2009). Isolation and Quantitative Determination of Ergosterol Peroxide in Various Edible Mushroom Species. Food Chem..

[26] Kadakal Ç., Artik N. (2008). Degradation Kinetics of Ergosterol in Tomato Paste Serum. Eur. Food Res. Technol..

[27] Weete J.D., Abril M., Blackwell M. (2010). Phylogenetic Distribution of Fungal Sterols. PLoS ONE.

[28] Brumfield K.M., Laborde S.M., Moroney J.V. (2017). A Model for the Ergosterol Biosynthetic Pathway in Chlamydomonas Reinhardtii. Eur. J. Phycol..

[29] Umaña M., Turchiuli C., Rosselló C., Simal S. (2021). Addition of a Mushroom By-Product in Oil-in-Water Emulsions for the Microencapsulation of Sunflower Oil by Spray Drying. Food Chem..

[30] Miller M.B., Haubrich B.A., Wang Q., Snell W.J., Nes W.D. (2012). Evolutionarily Conserved Δ25(27)-Olefin Ergosterol Biosynthesis Pathway in the Alga Chlamydomonas Reinhardtii. J. Lipid Res..

[31] Fang Y.-G., Khan Z., Hu F.-C., Su X.-H., Xing L.-X. (2025). Enhancement and Mechanism of Ergosterol Biosynthesis in Termite Ball Fungus Athelia Termitophila by Methyl Jasmonate. Curr. Issues Mol. Biol..

[32] Jordá T., Rozès N., Martínez-Pastor M.T., Puig S. (2023). The Yeast mRNA-Binding Protein Cth2 Post-Transcriptionally Modulates Ergosterol Biosynthesis in Response to Iron Deficiency. Biochim. Biophys. Acta (BBA) Gene Regul. Mech..

[33] Jordá T., Barba-Aliaga M., Rozès N., Alepuz P., Martínez-Pastor M.T., Puig S. (2022). Transcriptional Regulation of Ergosterol Biosynthesis Genes in Response to Iron Deficiency. Environ. Microbiol..

[34] Han Z., Zong Y., Zhang X., Gong D., Wang B., Prusky D., Sionov E., Xue H., Bi Y. (2023). Erg4 Is Involved in Ergosterol Biosynthesis, Conidiation and Stress Response in Penicillium Expansum. J. Fungi.

[35] Liang R., Xu K., Wang X., Wei W., Chen Q., Qin Z., Zeng W., Zhou J. (2024). Rational Design of Lanosterol 14α-Demethylase for Ergosterol Biosynthesis in *Saccharomyces cerevisiae*. 3 Biotech.

[36] Liu X., Jiang J., Yin Y., Ma Z. (2013). Involvement of FgERG4 in Ergosterol Biosynthesis, Vegetative Differentiation and Virulence in Fusarium Gra-Minearum. Mol. Plant Pathol..

[37] Khan M.S., Ahmad I., Cameotra S. (2013). Phenyl Aldehyde and Propanoids Exert Multiple Sites of Action towards Cell Membrane and Cell Wall Targeting Ergosterol in Candida Albicans. AMB Express.

[38] Sasidharan S., Nishanth K.S., Nair H.J. (2023). A Semi Purified Hydroalcoholic Fraction from Caesalpinia Bonduc Seeds Causes Ergosterol Biosynthesis Inhibition in Candida Albicans Resulting in Cell Membrane Damage. Front. Pharmacol..

[39] Yang S., Yan D., Li M., Li D., Zhang S., Fan G., Peng L., Pan S. (2022). Ergosterol Depletion under Bifonazole Treatment Induces Cell Membrane Damage and Triggers a ROS-Mediated Mitochondrial Apoptosis in Penicillium Expansum. Fungal Biol..

[40] Zhang K., Wang Q., Zhang N., Yu L., Lin Q., Zhou W. (2025). Inhibition Effect of 2-Ethylhexanol against *Aspergillus Flavus* and Aflatoxin B1 Mainly by Disrupting Cell Membrane and Downregulating Genes Related to Ergosterol Synthesis and Aflatoxins Global Regulator. Food Chem..

[41] Wei J., Bi Y., Xue H., Wang Y., Zong Y., Prusky D. (2020). Antifungal Activity of Cinnamaldehyde against Fusarium Sambucinum Involves Inhibition of Ergosterol Biosynthesis. J. Appl. Microbiol..

[42] Irshad M., Shreaz S., Manzoor N., Khan L.A., Rizvi M.M.A. (2011). Anticandidal Activity of Cassia Fistula and Its Effect on Ergosterol Biosynthesis. Pharm. Biol..

[43] Chang W., Zhang M., Li Y., Li X., Gao Y., Xie Z., Lou H. (2015). Lichen Endophyte Derived Pyridoxatin Inactivates Candida Growth by Interfering with Ergosterol Biosynthesis. Biochim. Biophys. Acta (BBA) General. Subj..

[44] Yamamoto E.S., De Jesus J.A., Bezerra-Souza A., Brito J.R., Lago J.H.G., Laurenti M.D., Passero L.F.D. (2020). Tolnaftate Inhibits Ergosterol Production and Impacts Cell Viability of *Leishmania* Sp.. Bioorganic Chem..

[45] Rather I.A., Sabir J.S.M., Asseri A.H., Wani M.Y., Ahmad A. (2022). Triazole Derivatives Target 14α–Demethylase (LDM) Enzyme in Candida Albicans Causing Ergosterol Biosynthesis Inhibition. J. Fungi.

[46] Choy H.L., Gaylord E.A., Doering T.L. (2023). Ergosterol Distribution Controls Surface Structure Formation and Fungal Pathogenicity. mBio.

[47] Trigos Á., Ortega-Regules A. (2002). Selective Destruction of Microscopic Fungi through Photo-Oxidation of Ergosterol. Mycologia.

[48] Ribeiro R.A., Godinho C.P., Vitorino M.V., Robalo T.T., Fernandes F., Rodrigues M.S., Sá-Correia I. (2022). Crosstalk between Yeast Cell Plasma Membrane Ergosterol Content and Cell Wall Stiffness under Acetic Acid Stress Involving Pdr18. J. Fungi.

[49] Chen J., Zhang M., Devahastin S. (2021). UV-C Irradiation-Triggered Nutritional Change of 4D Printed Ergosterol-Incorporated Purple Sweet Potato Pastes: Conversion of Ergosterol into Vitamin D2. LWT.

[50] Kaur M., Sharma S., Kaur R., Kaur J., Singh A. (2023). Effect of UV-B Irradiation on Bioconversion of Ergosterol to Vitamin D2 and Its Impact on Nutritional Properties of Oyster Mushroom. Int. J. Food Sci. Technol..

[51] Jasinghe V.J., Perera C.O. (2005). Distribution of Ergosterol in Different Tissues of Mushrooms and Its Effect on the Conversion of Ergosterol to Vitamin D2 by UV Irradiation. Food Chem..

[52] Perera C.O., Jasinghe V.J., Ng F.L., Mujumdar A.S. (2003). The Effect of Moisture Content on the Conversion of Ergosterol to Vitamin D in Shiitake Mushrooms. Dry. Technol..

[53] Sommer K., Hillinger M., Eigenmann A., Vetter W. (2023). Characterization of Various Isomeric Photoproducts of Ergosterol and Vitamin D2 Generated by UV Irradiation. Eur. Food Res. Technol..

[54] Chen J., Zhang M., Mujumdar A.S., Phuhongsunge P. (2022). 4D Printing Induced by Microwave and Ultrasound for Mushroom Mixtures: Efficient Conversion of Ergosterol into Vitamin D2. Food Chem..

[55] Perkowski J., Buśko M., Stuper K., Kostecki M., Matysiak A., Szwajkowska-Michałek L. (2008). Concentration of Ergosterol in Small-Grained Naturally Contaminated and Inoculated Cereals. Biologia.

[56] Barajas-Aceves M., Hassan M., Tinoco R., Vazquez-Duhalt R. (2002). Effect of Pollutants on the Ergosterol Content as Indicator of Fungal Biomass. J. Microbiol. Methods.

[57] Ruzicka S., Edgerton D., Norman M., Hill T. (2000). The Utility of Ergosterol as a Bioindicator of Fungi in Temperate Soils. Soil Biol. Biochem..

[58] Hippelein M., Rügamer M. (2004). Ergosterol as an Indicator of Mold Growth on Building Materials. Int. J. Hyg. Environ. Health.

[59] Saxena J., Munimbazi C., Bullerman L.B. (2001). Relationship of Mold Count, Ergosterol and Ochratoxin A Production. Int. J. Food Microbiol..

[60] Vella F.M., Calandrelli R., Del Barone A., Guida M., Laratta B. (2023). Rapid Evaluation of Ergosterol to Detect Yeast Contamination in Fruit Juices. Eur. Food Res. Technol..

[61] Friedrichs S.P., Doern C.D., Jamerson M.J., Korzun W.J. (2025). Analytical Performance of an Assay for Ergosterol, a Potential Biomarker for *Naegleria fowleri* in Cerebrospinal Fluid. Lab. Med..

[62] Gil-Ramírez A., Caz V., Martin-Hernandez R., Marín F.R., Largo C., Rodríguez-Casado A., Tabernero M., Ruiz-Rodríguez A., Reglero G., Soler-Rivas C. (2016). Modulation of Cholesterol-Related Gene Expression by Ergosterol and Ergosterol-Enriched Extracts Obtained from *Agaricus bisporus*. Eur. J. Nutr..

[63] Harmhan D.J. (1953). Studies on Absorption and Metabolism of Ergosteroll-Cl4. Arch. Biochem. Biophys..

[64] Machida K., Koseki Y., Kotani A., Yamamoto K., Miura T., Hakamata H. (2022). Simultaneous Determination of Deuterium-Labeled Ergosterol and Brassicasterol in Stroke-Prone Spontaneously Hypertensive Rats by Ultra-High Performance Liquid Chromatography-Electrospray Ionization-Tandem Mass Spectrometry. Anal. Methods.

[65] Ling T., Arroyo-Cruz L.V., Smither W.R., Seighman E.K., Martínez-Montemayor M.M., Rivas F. (2024). Early Preclinical Studies of Ergosterol Peroxide and Biological Evaluation of Its Derivatives. ACS Omega.

[66] Slominski A., Semak I., Zjawiony J., Wortsman J., Gandy M.N., Li J., Zbytek B., Li W., Tuckey R.C. (2005). Enzymatic Metabolism of Ergosterol by Cytochrome P450scc to Biologically Active 17α,24-Dihydroxyergosterol. Chem. Biol..

[67] Michaelw N. (1961). Oxidation of Ergosterol by Rat and Mouse Liver Mitochondria. Proc. Soc. Exp. Biol. Med..

[68] Zhang H., Firempong C.K., Wang Y., Xu W., Wang M., Cao X., Zhu Y., Tong S., Yu J., Xu X. (2016). Ergosterol-Loaded Poly(Lactide-Co-Glycolide) Nanoparticles with Enhanced in Vitro Antitumor Activity and Oral Bioavailability. Acta Pharmacol. Sin..

[69] Dong Z., Iqbal S., Zhao Z. (2020). Preparation of Ergosterol-Loaded Nanostructured Lipid Carriers for Enhancing Oral Bioavailability and Antidiabetic Nephropathy Effects. AAPS PharmSciTech.

[70] Yi C., Fu M., Cao X., Tong S., Zheng Q., Firempong C.K., Jiang X., Xu X., Yu J. (2013). Enhanced Oral Bioavailability and Tissue Distribution of a New Potential Anticancer Agent, *Flammulina velutipes* Sterols, through Liposomal Encapsulation. J. Agric. Food Chem..

[71] Yi C., Sun C., Tong S., Cao X., Feng Y., Firempong C.K., Jiang X., Xu X., Yu J. (2013). Cytotoxic Effect of Novel *Flammulina Velutipes* Sterols and Its Oral Bioavailability via Mixed Micellar Nanoformulation. Int. J. Pharm..

[72] Xu X., Yi C., Zhong H., Tong S., Cao X., Liu H., Fu M., Zhang H., Firempong C.K., Yu J. (2012). Enhanced Oral Bioavailability of a Sterol-Loaded Microemulsion Formulation of *Flammulina Velutipes*, a Potential Antitumor Drug. Int. J. Nanomed..

[73] Ang L., Yuguang L., Liying W., Shuying Z., Liting X., Shumin W. (2015). Ergosterol Alleviates Kidney Injury in Streptozotocin-Induced Diabetic Mice. Evid.-Based Complement. Altern. Med..

[74] Liu C., Zhao S., Zhu C., Gao Q., Bai J., Si J., Chen Y. (2020). Ergosterol Ameliorates Renal Inflammatory Responses in Mice Model of Diabetic Nephropathy. Biomed. Pharmacother..

[75] Dong Z., Li X., Wang X., Xu J., Xu W. (2025). Ergosterol from Edible Fungi: Enhancing Fatty Acid Oxidation via CPT1A to Protect against Diabetic Kidney Disease. Food Funct..

[76] Dong Z., Sun Y., Wei G., Li S., Zhao Z. (2019). Ergosterol Ameliorates Diabetic Nephropathy by Attenuating Mesangial Cell Proliferation and Extracellular Matrix Deposition via the TGF-Β1/Smad2 Signaling Pathway. Nutrients.

[77] Gil-Ramírez A., Ruiz-Rodríguez A., Marín F.R., Reglero G., Soler-Rivas C. (2014). Effect of Ergosterol-Enriched Extracts Obtained from *Agaricus bisporus* on Cholesterol Absorption Using an in Vitro Digestion Model. J. Funct. Foods.

[78] Kuwabara N., Ohta-Shimizu M., Fuwa F., Tomitsuka E., Sato S., Nakagawa S. (2022). Ergosterol Increases 7-Dehydrocholesterol, a Cholesterol Precursor, and Decreases Cholesterol in Human HepG2 Cells. Lipids.

[79] Hussein Zaki A., Haiying B., Mohany M., Al-Rejaie S.S., Abugammie B. (2024). The Effect Mechanism of Ergosterol from the Nutritional Mushroom Leucocalocybe Mongolica in Breast Cancer Cells: Protein Expression Modulation and Metabolomic Profiling Using UHPLC-ESI-Q. Saudi Pharm. J..

[80] Taank Y., Agnihotri N. (2025). Exploring the Anticancer Potential of Ergosterol and Ergosta-5,22,25-Triene-3-Ol against Colorectal Cancer: Insights from Experimental Models and Molecular Mechanisms. Biochem. Pharmacol..

[81] Han J., Sohn E.J., Kim B., Kim S., Won G., Yoon S., Lee J., Kim M.J., Lee H., Chung K. (2014). Ergosterol Reverses Multidrug Resistance in SGC7901/Adr Cells. Pharmazie.

[82] Han J., Sohn E.J., Kim B., Kim S., Won G., Yoon S., Lee J., Kim M.J., Lee H., Chung K. (2014). Upregulation of Death Receptor 5 and Activation of Caspase 8/3 Play a Critical Role in Ergosterol Peroxide Induced Apoptosis in DU 145 Prostate Cancer Cells. Cancer Cell Int..

[83] Shin M.-K., Sasaki F., Ki D.-W., Win N.N., Morita H., Hayakawa Y. (2021). Anti-Metastatic Effects of Ergosterol Peroxide from the Entomopathogenic Fungus Ophiocordyceps Gracilioides on 4T1 Breast Cancer Cells. J. Nat. Med..

[84] Bocachica-Adorno A.L., Aponte-Ramos A.Y., Rivera-Fuentes P.S., Espinosa-Ponce N.P., Arroyo-Cruz L.V., Ling T., Pérez-Ríos N., Rivas-Tumanyan S., Almodóvar-Rivera I.A., Barreto-Gamarra C. (2025). Ergosterol Peroxide Disrupts Triple-Negative Breast Cancer Mitochondrial Function and Inhibits Tumor Growth and Metastasis. Int. J. Mol. Sci..

[85] Wu H.-Y., Yang F.-L., Li L.-H., Rao Y.K., Ju T.-C., Wong W.-T., Hsieh C.-Y., Pivkin M.V., Hua K.-F., Wu S.-H. (2018). Ergosterol Peroxide from Marine Fungus Phoma Sp. Induces ROS-Dependent Apoptosis and Autophagy in Human Lung Adenocarcinoma Cells. Sci. Rep..

[86] Meza-Menchaca T., Poblete-Naredo I., Albores-Medina A., Pedraza-Chaverri J., Quiroz-Figueroa F.R., Cruz-Gregorio A., Zepeda R.C., Melgar-Lalanne G., Lagunes I., Trigos Á. (2020). Ergosterol Peroxide Isolated from Oyster Medicinal Mushroom, Pleurotus Ostreatus (Agaricomycetes), Potentially Induces Radiosensitivity in Cervical Cancer. Int. J. Med. Mushrooms.

[87] Deng S., Wang L., Tian S., Wu J., Lin Y., Wang H., Guo X., Han C., Ren W., Han Y.L. (2025). Thiazolidinedione-Based Structure Modification of Ergosterol Peroxide Provides Thiazolidinedione-Conjugated Derivatives as Potent Agents against Breast Cancer Cells through a PI3K/AKT/mTOR Pathway. Bioorganic Med. Chem..

[88] Tan W., Pan M., Liu H., Tian H., Ye Q., Liu H. (2017). Ergosterol Peroxide Inhibits Ovarian Cancer Cell Growth through Multiple Pathways. OncoTargets Ther..

[89] Zhang C.-C., Yin X., Cao C.-Y., Wei J., Zhang Q., Gao J.-M. (2015). Chemical Constituents from Hericium Erinaceus and Their Ability to Stimulate NGF-Mediated Neurite Outgrowth on PC12 Cells. Bioorganic Med. Chem. Lett..

[90] Li L., Zhu Y., Cheng W., Di F., Zhang J., Wang C. (2024). Efficacy of Ergosterol and Ergosterol Peroxide in Different Anti-Inflammatory Models—A Comparison Study. Food Agric. Immunol..

[91] Zhu R., Zheng R., Deng Y., Chen Y., Zhang S. (2014). Ergosterol Peroxide from Cordyceps Cicadae Ameliorates TGF-Β1-Induced Activation of Kidney Fibroblasts. Phytomedicine.

[92] Ikarashi N., Hoshino M., Ono T., Toda T., Yazawa Y., Sugiyama K. (2020). A Mechanism by Which Ergosterol Inhibits the Promotion of Bladder Carcinogenesis in Rats. Biomedicines.

[93] Yazawa Y., Ikarashi N., Hoshino M., Kikkawa H., Sakuma F., Sugiyama K. (2020). Inhibitory Effect of Ergosterol on Bladder Carcinogenesis Is Due to Androgen Signaling Inhibition by Brassicasterol, a Metabolite of Ergosterol. J. Nat. Med..

[94] Nowak R., Drozd M., Mendyk E., Lemieszek M., Krakowiak O., Kisiel W., Rzeski W., Szewczyk K. (2016). A New Method for the Isolation of Ergosterol and Peroxyergosterol as Active Compounds of Hygrophoropsis Aurantiaca and in Vitro Antiproliferative Activity of Isolated Ergosterol Peroxide. Molecules.

[95] Russo A., Cardile V., Piovano M., Caggia S., Espinoza C.L., Garbarino J.A. (2010). Pro-Apoptotic Activity of Ergosterol Peroxide and (22E)-Ergosta-7,22-Dien-5α-Hydroxy-3,6-Dione in Human Prostate Cancer Cells. Chem.-Biol. Interact..

[96] Zhou H.B., Feng L.J., Liang X.D.K., Zhou Z.X., Li M., Zhang J.L., Su G.H. (2024). Mechanism of Cordyceps Militaris in Gouty Nephropathy Explored Using Network Pharmacology and Molecular Docking Technology. Food Biosci..

[97] Wang H., Zhang T., Li Y., Wang S. (2018). Effects of Ergosterol on COPD in Mice via JAK3/STAT3/NF-κB Pathway. Inflammation.

[98] Tai C.-J., Choong C.-Y., Lin Y.-C., Shi Y.-C., Tai C.-J. (2016). The Anti-Hepatic Fibrosis Activity of Ergosterol Depended on Upregulation of PPARgamma in HSC-T6 Cells. Food Funct..

[99] Sillapachaiyaporn C., Chuchawankul S., Nilkhet S., Moungkote N., Sarachana T., Ung A.T., Baek S.J., Tencomnao T. (2022). Ergosterol Isolated from Cloud Ear Mushroom (Auricularia Polytricha) Attenuates Bisphenol A-Induced BV2 Microglial Cell Inflammation. Food Res. Int..

[100] Song W., Zhu L., Yang C., Su K., Miao Y., Hu J., Chen B., Li L., Cui X., Luo Y. (2025). Ergosterol Originated from Auricularia Auricula Attenuates High Fat Diet-Induced Obesity and Cognitive Impairment in Mice. Food Funct..

[101] Kou R.-W., Zhang R.-J., Xia B., Bai G.-Y., Liu G.-S., He Y.-Q., Gao J.-M., Tang J.-J. (2025). Ergosterol Regulates Visceral Hypersensitivity and Intestinal Motility in Irritable Bowel Syndrome Through Microbiota–Metabolites–AhR Signaling Axis. J. Agric. Food Chem..

[102] Cai D., Yan H., Liu J., Chen S., Jiang L., Wang X., Qin J. (2021). Ergosterol Limits Osteoarthritis Development and Progression through Activation of Nrf2 Signaling. Exp. Ther. Med..

[103] Yang H., Guo Y., Wang S., Lin K., Wang Y., Hou J., Cao J., Cheng Y., Cheng F., Yun S. (2024). A Novel Approach for Delivery of Ergosterol Within Ferritin Cage: Stability, Slow-Release Property, and Cholesterol-Lowering Effect After Simulated Gastrointestinal Digestion. Food Biophys..

[104] Sharma H., Chandra P. (2025). Development of Ergosterol Nanoliposome-Based Delivery System Pertaining Toxicity Evaluation and Therapeutic Potential for Alzheimer’s Disease. CNS Neurol. Disord. -Drug Targets.

[105] Wu M., Huang T., Wang J., Chen P., Mi W., Ying Y., Wang H., Zhao D., Huang S. (2018). Antilung Cancer Effect of Ergosterol and Cisplatin-Loaded Liposomes Modified with Cyclic Arginine-Glycine-Aspartic Acid and Octa-Arginine Peptides. Medicine.

[106] Tai K., Liu F., He X., Ma P., Mao L., Gao Y., Yuan F. (2018). The Effect of Sterol Derivatives on Properties of Soybean and Egg Yolk Lecithin Liposomes: Stability, Structure and Membrane Characteristics. Food Res. Int..

[107] Cui M., Wu W., Hovgaard L., Lu Y., Chen D., Qi J. (2015). Liposomes Containing Cholesterol Analogues of Botanical Origin as Drug Delivery Systems to Enhance the Oral Absorption of Insulin. Int. J. Pharm..

[108] Gao J., Tang L., Fu C., Cao Y., Liu H., Yin Y., Li Z., Zhu Y., Shu W., Zhang Y. (2025). A Nano-Strategy for Advanced Triple-Negative Breast Cancer Therapy by Regulating Intratumoral Microbiota. Nano Lett..

[109] De Planque M.R.R., Mendes G.P., Zagnoni M., Sandison M.E., Fisher K.H., Berry R.M., Watts A., Morgan H. (2006). Controlled Delivery of Membrane Proteins to Artificial Lipid Bilayers by Nystatin–Ergosterol Modulated Vesicle Fusion. IEE Proc. Nanobiotechnol..

[110] Studer A., Demarche S., Langenegger D., Tiefenauer L. (2011). Integration and Recording of a Reconstituted Voltage-Gated Sodium Channel in Planar Lipid Bilayers. Biosens. Bioelectron..

[111] Ding X., Wang H., Liu F., Wang Y., Gao L., Yang F., Liu D. (2025). Biomimetic RBC-Membrane-Cloaked Prussian Blue Nanoparticles for Synergistic Photothermal-Antibacterial Therapy and Accelerated Wound Healing. Mater. Des..

[112] Taheri S., Ahadi Z., Matta C.F., Ghanbarzadeh S., Shadman Lakmehsari M. (2023). The Effects of the Nature of the Sterol on the Properties and Stability of Niosome Bilayer Vesicles. J. Mol. Liq..

[113] Barani M., Reza Hajinezhad M., Sargazi S., Zeeshan M., Rahdar A., Pandey S., Khatami M., Zargari F. (2021). Simulation, In Vitro, and In Vivo Cytotoxicity Assessments of Methotrexate-Loaded pH-Responsive Nanocarriers. Polymers.

[114] Hajinezhad M.R., Fathi-karkan S., Roostaee M., Sargazi S., Mirinejad S., Sheervalilou R., Sargazi S., Barani M. (2025). Engineering pH-Responsive Niosomes with Ergosterol and CHEMS for Controlled Carfilzomib Release: Insights from in Vitro and in Vivo Studies. Drug Dev. Ind. Pharm..

[115] Barani M., Mirzaei M., Torkzadeh-Mahani M., Adeli-sardou M. (2019). Evaluation of Carum-Loaded Niosomes on Breast Cancer Cells:Physicochemical Properties, In Vitro Cytotoxicity, Flow Cytometric, DNA Fragmentation and Cell Migration Assay. Sci. Rep..

[116] Barani M., Torkzadeh-Mahani M., Mirzaei M., Nematollahi M.H. (2020). Comprehensive Evaluation of Gene Expression in Negative and Positive Trigger-Based Targeting Niosomes in HEK-293 Cell Line. Iran. J. Pharm. Res..

[117] Cheng J., Zhao H., Yao L., Li Y., Qi B., Wang J., Yang X. (2019). Simple and Multifunctional Natural Self-Assembled Sterols with Anticancer Activity-Mediated Supramolecular Photosensitizers for Enhanced Antitumor Photodynamic Therapy. ACS Appl. Mater. Interfaces.

[118] Zalnezhad S., Adeli-Sardou M., Roostaee M., Barani M., Mirzaei M., Sargazi G. (2025). Synergistic Anti-Cancer and Antimicrobial Effects of Ganoderma Lucidum and Lentinus Edodes Mushroom Extracts-Loaded Niosomes. J. Surfact Deterg..

[119] Rodero C.F., Fioramonti Calixto G.M., Cristina Dos Santos K., Sato M.R., Aparecido Dos Santos Ramos M., Miró M.S., Rodríguez E., Vigezzi C., Bauab T.M., Sotomayor C.E. (2018). Curcumin-Loaded Liquid Crystalline Systems for Controlled Drug Release and Improved Treatment of Vulvovaginal Candidiasis. Mol. Pharm..

[120] Rahimi F., Amoabediny G., Sabahi H., Zandieh-Doulabi B. (2022). Fungal Infected Adipose Stem Cells: The Effects of Novel Lipo-Niosome Nanoparticles Loaded with Amphotericin B and Thymus Essential Oil. Cell J..

[121] Du X., Huang X., Wang L., Mo L., Jing H., Bai X., Wang H. (2022). Nanosized Niosomes as Effective Delivery Device to Improve the Stability and Bioaccessibility of Goat Milk Whey Protein Peptide. Food Res. Int..

